# Safeguarding a Flagship Species: Integrated Surveillance of Cross‐Species Pathogen Transmission in Giant Panda Ecosystems

**DOI:** 10.1002/ece3.73260

**Published:** 2026-03-14

**Authors:** Xiaoli Sun, Yi Peng, Xiaoye Hao, Rong Dong, Zhilin Wang, Le Wang, Chengdong Wang, Xiangdong Wu, Zheng Chen, Wenbo Zhang, Xiaoli Tang

**Affiliations:** ^1^ College of Animal Science and Technology Jiangxi Agricultural University Nanchang China; ^2^ Research Center for Qinling Giant Panda Shaanxi Academy of Forestry Xi'an China; ^3^ Jiangxi Provincial Key Laboratory of Conservation Biology, College of Forestry Jiangxi Agricultural University Nanchang China; ^4^ Key Laboratory of Southwest China Wildlife Resources Conservation (Ministry of Education) China West Normal University Nanchong China; ^5^ Chengdu Research Base of Giant Panda Breeding Chengdu China

**Keywords:** disease cross‐species transmission, domestic‐wild animal interface, Giant panda, metagenomics, pathogen surveillance

## Abstract

Emerging infectious diseases, driven by increasing interactions among humans, wildlife, and livestock, pose an escalating threat to global health, biodiversity, and economies. As a flagship endangered species, the giant panda ( *Ailuropoda melanoleuca* ) plays a pivotal role in biodiversity conservation in China. This review synthesizes current knowledge on pathogens threatening giant panda health, including viruses, bacteria, and parasites alongside their potential transmission pathways within nature reserves. We emphasize the roles of domesticated animals, sympatric wildlife, and ectoparasites as reservoir hosts or vectors. Special focus is placed on cross‐species transmission dynamics and the critical need for integrated monitoring systems utilizing metagenomics and viromics. We propose a framework for establishing early warning systems and surveillance networks at the domestic‐wild animal interface to enhance pathogen detection, disease prevention, and biodiversity conservation.

## Graphical abstract

Emerging infectious diseases increasingly threaten giant pandas and cohabiting species. This review analyzes pathogen transmission risks from domestic animals, wildlife, and vectors and proposes an integrated genomic surveillance framework for early warning and biodiversity conservation.

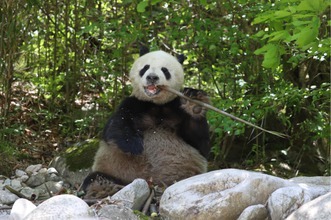

## 1 Introduction

In recent decades, emerging infectious diseases, such as plague, Middle East Respiratory Syndrome, and COVID‐19, have presented unprecedented challenges to wildlife conservation, public health, biodiversity, and socio‐economic development. These threats stem from complex factors, including pathogen evolution, climate change, land‐use alterations, economic development, and human population expansion (Tang et al. 2020). The degradation and fragmentation of natural habitats have intensified interactions between humans, domestic animals, wildlife, and vectors, creating ideal conditions for cross‐species transmission of infectious agents.

Research indicates that wild mammals harbor at least 878 viral species, including 170 with zoonotic potential, and livestock carry 288 viruses, of which 117 are zoonotic (Shivaprakash et al. 2021). Notably, pathogen transmission at the domestic–wild animal interface is frequently bidirectional (Miller et al. 2013). It is estimated that over 70% of zoonotic diseases originate from wildlife (Qin et al. 2010). Furthermore, approximately 1.7 million undiscovered viral species exist in wildlife globally, with 540,000–850,000 potentially capable of infecting humans directly or via domestic animals (Carroll et al. 2018). Conversely, infectious diseases have also emerged as a primary threat to wildlife survival. Human activities, including animal farming and global trade, exacerbate pathogen spread. Notably, a large proportion of livestock pathogens (77%) and an even higher proportion of carnivore pathogens (91%) can infect multiple hosts, including wildlife (Miller et al. 2013). For instance, during 2016–2017, the Peste‐des‐petits‐ruminants' virus spilled over from livestock to the critically endangered Saiga antelope ( *Saiga tatarica* ), causing 80% mortality (Kuiken and Cromie 2022). Since 2007, African Swine Fever Virus (ASFV) has spread across Europe and Asia through pig and pork trade, subsequently spilling over to wild boar ( *Sus scrofa* ) populations and threatening Southeast Asia's 11 endemic wild pig species (Luskin et al. 2020).

Eradicating livestock‐derived pathogens once established in wildlife populations remains extremely challenging. Therefore, preventing pathogen spillover at the domestic and wildlife interface is essential for wildlife conservation, public health, and economic stability (Miller et al. 2013). However, current understanding of disease dynamics and transmission mechanisms at this interface remains limited. The absence of effective early warning systems and diagnostic tools hinders the control of emerging infectious diseases. This underscores the urgent need for enhanced surveillance, risk assessment, and control strategies for these diseases in ecosystems shared by domestic and wild species.

**FIGURE 1:**
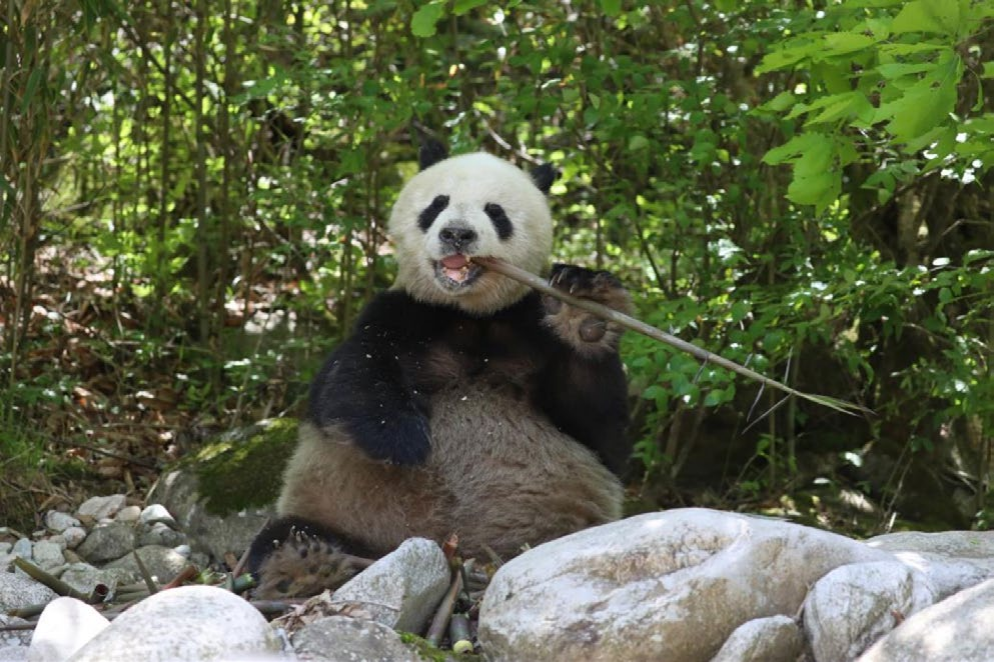
Wild giant panda ( *Ailuropoda melanoleuca* ). Photographed by Guangping Huang. Used with permission.

The giant panda, an endemic and endangered species in China, is a global flagship species for biodiversity conservation (Figure 1) (Wei et al. 2020). Due to successful conservation efforts, the IUCN reclassified its status from “Endangered” to “Vulnerable” in 2016 (Swaisgood et al. 2016). According to the Fourth National Survey on Giant Pandas, an estimated 1864 wild individuals are confined to six mountain ranges: the Qinling, Minshan, Qionglai, Daxiangling, Xiaoxiangling, and Liangshan Mountains (Hu et al. 2021). In 2021, the Giant Panda National Park was officially established across primary panda habitats in Sichuan, Shaanxi, and Gansu provinces, covering 22,000 km^2^. The park established ecological corridors connecting 13 local panda populations, thereby protecting 71.9% (1340 out of 1864) of the wild giant pandas nationwide. This park harbors numerous endemic, rare, and endangered animals and is critical for Asian biodiversity conservation. Significant human populations reside within and around these reserves, with villages often overlapping reserve boundaries, some situated near core panda habitats (Callan et al. 2020). This proximity facilitates direct and indirect contact among residents, domestic animals (livestock, dogs, cats), giant pandas, and sympatric wildlife, substantially increasing pathogen transmission risks. Documented cases include giant panda deaths from Canine Distemper Virus (CDV) transmitted by domestic dogs (Hvistendahl 2015), the first reported cases of *Hepatozoon* sp. infection and human‐origin pH1N1 influenza infection in pandas (Martelli et al. 2019), and the potential transmission of *Enterocytozoon bieneusi* from pandas to humans (Li et al. 2018). These incidents highlight the critical need to establish baseline data on pathogens carried by giant pandas, sympatric wildlife, domestic animals, and vectors within panda reserves. Research into transmission mechanisms and the creation of a wildlife pathogen community database are essential for supporting disease prevention and control efforts for giant pandas and other endangered wildlife, ultimately contributing to species conservation, biodiversity maintenance, and human health. This review first comprehensively summarizes currently reported pathogens (viruses, bacteria, parasites) threatening giant panda health, detailing their prevalence, spatiotemporal distribution, morbidity, and mortality. Second, it synthesizes current knowledge on transmission pathways involving these pathogens, elucidating the impact of pathogens carried by sympatric animals, domestic animals, and vectors on giant pandas.

## 2 Pathogens Threatening the Health of Giant Pandas

Given the giant panda's vulnerable conservation status, the presence of any pathogen warrants consideration for its clinical and conservation management implications. Although some pathogens may be asymptomatic in healthy captive individuals, they can cause severe consequences in juveniles, elderly animals, or immunocompromised individuals, disproportionately affecting small and isolated populations (Smith et al. 2006; Yu et al. 2019). Moreover, co‐infections may potentiate the pathogenic effects of individual microorganisms (Simpson et al. 2013). Currently, the threat of infectious diseases within the giant panda's natural habitat is substantial. Panda reserves are often intricately linked with human settlements. For example, Wolong Nature Reserve protects approximately 2000 km^2^ but is surrounded by 1436 households (Callan et al. 2020). Liziping National Nature Reserve, a key site for panda translocation and reintroduction, is surrounded by villages housing over 7000 residents (Liu et al. 2022). Road construction, human settlements near panda habitat, and the presence of domestic pets and livestock directly increase the livestock–wildlife interface, promoting disease transmission. Furthermore, habitat loss and fragmentation concentrate pandas within remnant bamboo forests (Zhu et al. 2011). Concurrently, panda density has increased fourfold as populations have grown (Zhang et al. 2008), elevating pathogen prevalence and infection intensity within panda populations and presenting unprecedented challenges for disease control. While the establishment of panda reserves protects this flagship species and benefits sympatric mammals through umbrella and ecosystem‐wide conservation effects (Tang et al. 2022), the broad host range of most pathogens means these sympatric wildlife species themselves harbor and transmit pathogens, posing a potential threat for disease outbreaks. Additionally, the current wild giant panda population is fragmented into 33 isolated subpopulations. Small, isolated populations face increased risks of inbreeding depression, potentially leading to reduced disease resistance and insufficient immunity against pathogens (Wang et al. 2018; Kong et al. 2021). Alarmingly, fifteen subpopulations face a > 90% extinction risk. Translocation conservation and reintroduction programs are crucial for panda population maintenance but also directly facilitate disease transmission between regions. Consequently, infectious diseases, including viral, bacterial, and parasitic infections, can rapidly spread among wildlife, domestic animals, and even humans within panda reserves, causing large‐scale outbreaks and mortality, posing significant threats to human health and biodiversity.

To systematically evaluate the infection status of pathogenic agents (including parasites, bacteria, and viruses) in giant pandas, this section conducted a comprehensive retrieval and screening of relevant published literature, followed by a systematized analysis of pathogen‐related information. The PubMed, Web of Science, and China National Knowledge Infrastructure (CNKI) databases were searched without imposing restrictions on publication dates (up to December 2025). The search strategy employed combinations of subject terms, pairing any of the terms “parasite,” “bacteria,” “virus,” or “pathogen” with any of the terms “giant panda,” “giant panda habitat,” “giant panda ecosystem,” or “giant panda reserve”. The scope of this review did not encompass gray literature, including, but not limited to, conference abstracts and unpublished data. A preliminary search yielded 922 articles. Following rigorous screening, 870 studies were excluded because of the following: (1) not focusing on giant pandas (*n* = 627), (2) no related to pathogens (*n* = 176), (3) non‐original research articles (e.g., reviews, editorials; *n* = 33), and (4) lack of detailed infection data (*n* = 34). Ultimately, 52 studies were included in the subsequent comprehensive analysis.

### 2.1 Viral Diseases

Although clinical data on viral infections in giant pandas remain limited, several viruses have been associated with disease and mortality in both captive and wild individuals. These include canine distemper virus (CDV), canine parvovirus (CPV), feline panleukopenia virus (FPV), adenovirus, coronavirus, parainfluenza virus, rabies virus, circovirus, and influenza viruses (Qin et al. 2010; Smith et al. 2006) (Table 1 and Table S1). Among these, CDV, CPV, FPV, and Canine Coronavirus have been directly linked to morbidity and mortality in giant pandas (Zhao, Hu, et al. 2023; Yipeng et al. 2017). CDV poses the most severe threat to captive pandas. During an outbreak in 2014–2015, five of six infected pandas died at the Shaanxi Rare Wildlife Rescue and Breeding Center (Feng et al. 2016). Giant panda habitats in Shaanxi, Sichuan, and Gansu provinces border three CDV high‐prevalence areas; any CDV outbreak in these regions could endanger panda populations (Yipeng et al. 2017). Rotavirus causes refractory diarrhea and chronic malnutrition syndrome in weaned cubs, parvovirus causes viral enteritis, and Influenza A virus also impacts panda health. The multi‐host nature of these pathogens threatens not only pandas but also other wildlife and humans. In 2023, a panda‐derived FPV strain causing severe or fatal symptoms was isolated; this strain could infect cell lines from other mammalian species, including humans (Zhao, Hu, et al. 2023). Beyond recognized viruses, numerous undiscovered viruses likely exist. Viral metagenomics has recently facilitated the discovery of numerous animal viruses, providing insights into virome composition and aiding in identifying etiological agents, monitoring zoonoses, and identifying novel and emerging viruses. Using viromics techniques, novel picornaviruses, anelloviruses (19 distinct variants), and papillomaviruses have been detected in giant pandas (Zhang et al. 2017). While representatives of these virus families were also detected in healthy pandas, a higher proportion was found in diseased individuals, suggesting a potential association with illness. Viral presence does not necessarily indicate disease but may represent a degree of symbiotic interaction between the virus and host (Virgin 2014). However, the stability of this virome–host association is uncertain; waning host immunity may increase viral replication (Li et al. 2013), leading to higher viral loads in immunocompromised pandas and further deteriorating their condition (Zhao, Hu, et al. 2023). Nevertheless, the transmission pathways, host adaptation variations, pathogenicity, and distribution patterns among sympatric animals of these viruses require further investigation.

**TABLE 1:** Viral infections in Giant pandas.

Category	Pathogen	Prevalence	Sample type	Detection method	Management status	Sampling sites	Sampling time	References
Alphainfluenzavirus	Influenza A virus subtype H1N1	100% (1/1 sample)	Nasal swab, blood	RT‐PCR, Hemagglutination test, Electron microscopy observation	Captive	Ya'an Giant Panda Protection and Research Center, Sichuan	2009	Li et al. (2014)
Protoparvovirus	Feline panleukopenia virus	99% (194/195 samples)	Feces	NPCR	Captive	Chengdu Giant Panda Breeding and Research Base	2020.04–2020.05	Min et al. (2022)
	Feline panleukopenia virus	100% (4/4 samples)	Feces	PCR, Illumina novaseq sequencing, Electron microscopy observation	Captive	Chengdu Giant Panda Breeding and Research Base	2020	Yang et al. (2023)
Respirovirus	Canine parainfluenza virus	0 (0/92 samples)	Blood	Hemagglutination inhibition test	Captive	Sichuan, Chengdu, Beijing	1994–2005	Qin et al. (2010)
Coronavirus	Canine Coronavirus	37.5% (3/8 samples)	Blood	Serum neutralization test	Captive	Wolong Nature Reserve	1992.02–1992.06	Susan et al. (1994)
	Canine Coronavirus	12.9% (8/62 samples)	Blood	Serum neutralization test	Captive	Sichuan Zoo and Reserve	N/A	Qiao et al. (2004)
	Canine Coronavirus	0 (0/92 samples)	Blood	Serum neutralization test	Captive	Sichuan, Chengdu, Beijing	1994–2005	Qin et al. (2010)
Varicellovirus	Canine Herpesvirus	0 (0/8 samples)	Blood	Serum neutralization test	Captive and wild	Wolong Nature Reserve	1992.02–1992.06	Susan et al. (1994)
Morbillivirus	Canine Distemper Virus	0 (0/8 samples)	Blood	Dot‐ELISA, Serum neutralization test	Wild	Foping National Nature Reserve	2013–2015	Yipeng et al. (2017)
	Canine Distemper Virus	27% (6/22 giant pandas)	Nasal swab, tissue	RT‐PCR	Captive	Shaanxi Rare Wildlife Rescue and Research Center	2014.12–2015.03	Feng et al. (2016)
	Canine Distemper Virus	9% (9/92 samples)	Blood	Serum neutralization test	Captive	Sichuan, Chengdu, Beijing	1994–2005	Qin et al. (2010)
	Canine Distemper Virus	80% (4/5 samples)	Feces, blood	Antigen diagnosis, real‐time RT‐PCR	Captive	Shaanxi Rare Wildlife Rescue and Breeding Research Center	2014.12–2015.03	Zhao et al. (2017)
	Canine Distemper Virus	25% (2/8 samples)	Blood	Serum neutralization test	Captive	Wolong Nature Reserve	1992.02–1992.06	Susan et al. (1994)
	Canine Distemper Virus	84% (37/44 samples)	Blood	Serum neutralization test	Captive	Chengdu Giant Panda Base	1998–2003	Loeffler et al. (2007)
Parvovirus	Canine Parvovirus	2.78% (1/36 samples)	Feces	PCR, Sanger sequencing	Captive	Sichuan, China	2011.11–2012.05	Ling, Shao‐lin, Shi‐jie, et al. (2013)
	Canine Parvovirus	50% (4/8 samples)	Feces	PCR, colloidal gold test strip	Wild	Wolong Nature Reserve	2020.11	Zhou et al. (2025)
	Canine Parvovirus	75% (6/8 samples)	Blood	Hemagglutination inhibition test	Captive	Wolong Nature Reserve	1992.02–1992.06	Susan et al. (1994)
	Canine Parvovirus	90% (40/44 samples)	Blood	Hemagglutination inhibition test	Captive	Chengdu Giant Panda Base	1998–2003	Loeffler et al. (2007)
	Canine Parvovirus	58% (54/92 samples)	Blood	Hemagglutination inhibition test	Captive	Sichuan, Chengdu, Beijing	1994–2005	Qin et al. (2010)
	Canine Parvovirus	15.3% (8/52 samples)	Feces	PCR	Captive	Chengdu Research Base of Giant Panda Breeding	N/A	Ling, Shao‐lin, Zhi‐he, et al. (2013)
Mastadenovirus	Canine adenovirus (CAV‐1)	28% (26/92 samples)	Blood	Serum neutralization test	Captive	Sichuan, Chengdu, Beijing	1994–2005	Qin et al. (2010)
	Canine adenovirus (CAV‐2)	50% (4/8 samples)	Blood	Serum neutralization test	Captive	Wolong Nature Reserve	1992.02–1992.06	Susan et al. (1994)
Papillomavirus	Papillomavirus	23% (3/13 samples)	Nasopharyngeal secretions	PCR, viral metagenomics	Captive and wild	Tangjiahe, Chengdu Base	2014–2015	Zhang et al. (2017)
Varicellovirus	Pseudorabies Virus	0 (0/8 samples)	Blood	Serum neutralization test	Captive and wild	Wolong Nature Reserve	1992.02–1992.06	Susan et al. (1994)
Anellovirus	Anellovirus	100% (12/12 samples)	Blood	PCR, Viral metagenomics	Captive and wild	Tangjiahe, Chengdu Base	2014–2015	Zhang et al. (2017)
Circovirus	Circovirus	30% (4/13 samples)	Feces	PCR, Viral metagenomics	Captive and wild	Tangjiahe, Chengdu Base	2014–2015	Zhang et al. (2017)
	Circovirus	25% (3/12 samples)	Blood	PCR, metagenomic sequencing	Captive	Qinling Giant Panda Research Center, Shaanxi	2019.06	Dai et al. (2021)
	Circovirus	100% (1/1 sample)	Blood	Viral metagenomics	Captive	Chengdu Giant Panda Breeding and Research Base, Sichuan	2015.04	Wang et al. (2023)

*Note:* Column Definitions: Category: Taxonomic family or genus of the virus; Pathogen: Specific viral species; Prevalence: Percentage of positive samples relative to the total number of samples tested; Sample Type: Samples used for detection; Detection Method: Primary technique employed for pathogen identification; Management Status: Indication of the host (giant panda) as captive or wild; Sampling Sites: Geographical location or institution where samples were collected; Sampling Time: Year or period when samples were collected; References: Corresponding to the source of the study.

Abbreviations: Dot‐ELISA, Dot Enzyme‐Linked Immunosorbent Assay; N/A, not available; PCR, polymerase chain reaction; nPCR, nested polymerase chain reaction; RT‐PCR, reverse transcription polymerase chain reaction.

### 2.2 Bacterial Infections and Antimicrobial Resistance

Gastrointestinal bacterial infections are considered a leading cause of mortality in giant pandas, as alterations in gut microbial community can lead to digestive disorders and the development of underlying diseases (Wei et al. 2015). Common bacterial pathogens include *Escherichia coli* , *Klebsiella pneumoniae* , *Campylobacter jejuni* , *Arizona* spp., *Pseudomonas aeruginosa* , *Clostridium* spp., and *Proteus mirabilis* (Tables 2 and S1) (Yan et al. 2024). The widespread use of antibiotics has led to an increased prevalence of antimicrobial resistance (AMR). Antibiotic resistance genes (ARGs) can be transmitted via mobile genetic elements (MGEs) such as plasmids and integrons, facilitating horizontal gene transfer among bacteria. Genes like *tolC*, *mepA*, and *mdtA* have been implicated in multi‐drug resistance in giant panda‐associated isolates, leading to the emergence of multidrug‐resistant bacteria and posing significant risks to panda health (Yan et al. 2024). In 2015, a giant panda at Shanghai Wildlife Park died from infection with multidrug‐resistant bacteria, such as *E. coli* , *P. aeruginosa* , and *Enterococcus faecalis* . The *E. coli* strain SH‐YH‐DH carried a self‐transmissible plasmid encoding multiple resistance genes (*bla*CTX‐M‐55, *bla*TEM‐1, *sul1*, *floR*, *strB*, *aac*(*6*′)*Ib*, *tetA/R*) and exhibited resistance to gentamicin, cefotaxime, ampicillin, chloramphenicol, trimethoprim‐sulfamethoxazole, and tetracycline (Zhou et al. 2018). The giant panda gut microbial community is a significant reservoir of ARGs, including high‐risk resistance genes (Cai et al. 2024). Analysis of fresh fecal samples from captive pandas at the Chengdu Research Base of Giant Panda Breeding using High‐Throughput Quantitative Polymerase Chain Reaction detected 47 ARGs and 8 MGEs among isolated *K. pneumoniae* strains; some strains carried multiple ARGs and MGEs, highlighting the importance of monitoring and researching AMR in wildlife populations (Yan et al. 2024). Furthermore, virulence genes (e.g., *magA*, *uge*, *ycf*, *entB*, *kpn*, *allS*, *wabG*, *mrk*, *iro*) and heavy metal tolerance genes are enriched in the panda microbiota, contributing to various symptoms and aiding bacterial immune evasion through different mechanisms (Yan et al. 2024). Compared to wild pandas, captive populations exhibit significantly higher relative abundances of high‐risk ARGs, virulence genes, and heavy metal tolerance genes (Cai et al. 2024; Guo et al. 2019). If captive individuals are reintroduced into the wild, this could have catastrophic consequences for wild panda populations, other wildlife, and ecosystems (Guo et al. 2019). Besides the impact of captive‐origin pathogens on wild populations, pathogens from wild populations also threaten captive pandas. In the 1980s, some captive pandas died from hemorrhagic enteritis caused by enteroinvasive *E. coli* O152 introduced by a rescued wild panda. Moreover, ARGs found in giant pandas and humans show close genetic similarity (Deng et al. 2024), emphasizing the significant impact of human activities on ARG prevalence in natural environments and the potential health risks ARGs pose to pandas. The environment is a key factor influencing AMR in the panda gut microbiome, as multiple ARGs and MGEs are shared between the gut and the habitat environment. In‐depth research on the distribution of gut microbiota and resistance genes among sympatric animals of the giant panda is crucial for species conservation efforts (Cai et al. 2024).

**TABLE 2:** Bacterial infections in Giant pandas.

Category	Pathogen	Prevalence	Sample type	Detection method	Management status	Sampling sites	Sampling time	References
*Raoultella*	*Raoultella ornithinolytica*	7% (9/128 samples)	Feces	Microscopic examination, 16S rDNA, biochemical identification	Captive	Dujiangyan Base, Ya'an Bifengxia Base and Chengdu Base	2017–2019	Yan et al. (2023)
*Staphylococcus*	*Staphylococcus epidermidis*	41.7% (10/24 giant pandas)	Vaginal swab	Microscopic examination, biochemical identification, BD Phoenix‐100	Captive	Ya'an Bifengxia	2014.03–2014.05	Yang et al. (2016)
*Clostridium*	*Clostridium perfringens*	61.29% (19/31 samples)	Feces	16S rRNA	Captive	China Conservation and Research Center for the Giant Panda Sichuan	N/A	Deng et al. (2025)
*Enterobacter*	*Enterobacter* sp.	40.96% (68/166 samples)	Feces	Microscopic examination, MALDI‐TOF‐MS, 16S rDNA	Captive	N/A	2018.05–2018.06	Wang, Zhang, et al. (2022)
*Enterococcus*	*Enterococcus* sp.	70.48% (117/166 samples)	Feces	Microscopic examination, MALDI‐TOF‐MS, 16S rDNA	Captive	N/A	2018.05–2018.06	Wang, Zhang, et al. (2022)
*Bacteroides*	*Bacteroides fragilis*	100% (1/1 sample)	vaginal Swab	Microscopic examination, 16S rRNA gene sequencing, biochemical identification	Cvaptive	Chengdu Research Base of Giant Panda Breeding	2019.09	Yue et al. (2024)
*Escherichia*	*Escherichia coli*	100% (166/166 samples)	Feces	Microscopic examination, MALDI‐TOF‐MS, 16S rDNA	Captive	N/A	2018.05–2018.06	Wang, Zhang, et al. (2022)
	*Escherichia coli*	100% (100/100 giant pandas)	Feces	Biochemical identification, 16S rDNA	Captive	Chengdu Research Base of Giant Panda Breeding	2020.07–2021.03	Li et al. (2024)
	*Escherichia coli*	33.3% (8/24 giant pandas)	Vaginal swab	Microscopic examination, biochemical identification, BD Phoenix‐100 automated microbiology system	Captive	Ya'an Bifengxia	2014.03–2014.05	Yang et al. (2016)
	*Escherichia coli*	100% (97/97 samples)	Feces, vaginal secretions	Biochemical identification	Captive	Bifengxia Base	2010–2011	Wang, Yan, et al. (2013)
	*Escherichia coli*	100% (1/1 sample)	Urine	16S rDNA	Captive	China Conservation and Research Center for the Giant Panda Dujiangyan	2017.05	(Wenwen et al. 2019)
	*Escherichia coli*	100% (22/22 samples)	Feces	16S rDNA	Captive	Dujiangyan Base	N/A	Hongyan et al. (2025)
	*Escherichia coli*	100% (100/100 samples)	Feces	Multiplex PCR	Captive	Chengdu Research Base of Giant Panda Breeding	2022.03–2022.04	Liu, Zheng, et al. (2023)
*Klebsiella*	*Klebsiella pneumoniae*	69.88% (116/166 samples)	Feces	Microscopic examination, MALDI‐TOF‐MS, 16S rDNA	Captive	N/A	2018.05–2018.06	Wang, Zhang, et al. (2022)
	*Klebsiella pneumoniae*	23% (42/182 samples)	Feces	Microscopic examination, 16S rDNA, biochemical identification	Captive	Chengdu Research Base of Giant Panda Breeding	2020	Yan et al. (2022)
	*Klebsiella pneumoniae*	4.2% (1/24 giant pandas)	Vaginal swab	Microscopic examination, biochemical identification, BD Phoenix‐100 automated microbiology system	Captive	Ya'an Bifengxia	2014.03–2014.05	Yang et al. (2016)
	*Klebsiella pneumoniae*	90% (90/100 samples)	Feces	Multiplex PCR	Captive	Chengdu Research Base of Giant Panda Breeding	2022.03–2022.04	Liu, Zheng, et al. (2023)
	*Klebsiella pneumoniae*	32.8% (42/128 samples)	Feces	Microscopic examination, 16S rDNA, biochemical identification	Captive	Dujiangyan Base, Ya'an Bifengxia Base and Chengdu Base	2017–2019	Yan et al. (2023)
	*Klebsiella pneumoniae*	59.02% (90/153 samples)	Feces	16S rDNA	Captive	Chengdu Giant Panda Breeding Base	2018.05–2018.12	Feng et al. (2022)
*Neisseria*	*Neisseria gonorrhoeae*	71.4% (10/14 samples)	Vaginal secretions	Metagenomic sequencing	Captive	Wolong, Dujiangyan, Ya'an Bifengxia, Ningbo Youngor Zoo	2018.06–2018.08	Zhang et al. (2020)
*Proteus*	*Proteus mirabilis*	4.2% (1/24 giant pandas)	Vaginal swab	Microscopic examination, biochemical identification, BD Phoenix‐100 automated microbiology system	Captive	Ya'an Bifengxia	2014.03–2014.05	Yang et al. (2016)
	*Proteus mirabilis*	30% (30/100 samples)	Feces	Multiplex PCR	Captive	Chengdu Research Base of Giant Panda Breeding	2022.03–2022.04	Liu, Zheng, et al. (2023)
	*Proteus mirabilis*	11.5% (17/148 samples)	Feces	Microscopic examination, 16S rRNA	Captive	Multiple Locations in Sichuan	2024.1.18–2024.7.6	Yizhou et al. (2025)
*Enterococcus*	*Enterococcus faecium*	93% (28/30 samples)	Saliva	Microscopic examination, 16S rDNA	Captive	Chengdu Research Base of Giant Panda Breeding	N/A	Liu, Huang, et al. (2023)
*Aeromonas*	*Aeromonas veronii*	100% (4/4 samples)	Organ tissues	Microscopic examination, 16S rRNA and gyrB gene sequencing, biochemical identification	Captive	Chengdu Research Base of Giant Panda Breeding	2020	(Su et al. 2023)
*Peptostreptococcus*	*Peptostreptococcus* sp.	12.5% (3/24 giant pandas)	Vaginal swab	Microscopic examination, biochemical identification, BD Phoenix‐100 automated microbiology system	Captive	Ya'an Bifengxia	2014.03–2014.05	Yang et al. (2016)
*Shigella*	*Shigella* sp.	62% (17/27 samples)	Feces	Microscopic examination, 16S rRNA sequencing, serological test	N/A	Wolong Giant Panda Protection Base	N/A	Ren et al. (2017)

*Note:* Column Definitions: Category: Taxonomic family or genus of the bacterium; Pathogen: Specific bacterial species or strain; Prevalence: Percentage of positive samples or giant pandas relative to the total number of samples or giant pandas tested; Sample Type: Samples used for detection; Detection Method: Primary technique employed for pathogen identification; Management Status: Indication of the host (giant panda) as captive or wild; Sampling Sites: Geographical location or institution where samples were collected; Sampling Time: Year or period when samples were collected; References: Corresponding to the source of the study.

Abbreviations: 16S rDNA/rRNA, 16S ribosomal DNA/RNA gene sequencing; BD Phoenix‐100, BD Phoenix‐100 Automated Microbiology System; MALDI‐TOF‐MS, Matrix‐assisted laser desorption/ionization time‐of‐flight mass spectrometry; PCR, polymerase chain reaction; Multiplex PCR, Multiplex polymerase chain reaction; N/A, not available.

### 2.3 Parasitic Infections

Parasitic diseases have long been recognized as major threats to the survival of wild giant pandas (Zhang et al. 2008). A retrospective analysis from 1971 to 2005 showed that parasitic infections accounted for over 50% of deaths in free‐ranging pandas. To date, at least 35 parasite species have been documented in pandas, belonging to nematodes (six species), trematodes (one species), cestodes (two species), protozoans (nine species), and ectoparasites (thirteen ticks, two mites, two fleas) Li et al. 2020; Table 3 and Table S1. Clinically significant cases commonly involve *Baylisascaris schroederi* ( *B. schroederi* , ascarid roundworm), *Chorioptes panda* (mange mite), and Ixodid ticks (Wang et al. 2018). *B. schroederi* has the highest prevalence (7%–88%) and mortality rate (50%) and is considered the primary cause of parasitic mortality (Zhang et al. 2008; Wang et al. 2018). Its larvae can migrate through visceral tissues, while adults can cause intestinal obstruction, pancreatitis, and hemorrhagic enteritis. One individual was found to harbor over 3200 adult worms (Zhang et al. 2010). Other prevalent endoparasites include *Ogmocotyle sikae* (trematode, prevalence 0.5%–100%), *Ancylostoma ailuropodae* (hookworm, prevalence 93.3%), *Enterocytozoon bieneusi* (microsporidian, zoonotic, prevalence 8.7%–34.5%), *Toxoplasma gondii* (*T. gondii*) (protozoan, zoonotic, prevalence 100%), and *Hepatozoon* sp. (protozoan, prevalence 73.9%). *A. ailuropodae* inhabits the small intestine, causing intestinal mucosal hemorrhage and inflammation (Xie et al. 2017). *O. sikae* also resides in the small intestine, leading to multiple mucosal hemorrhagic spots and digestive dysfunction (Li et al. 2020). *Cryptosporidium* spp. and *Enterocytozoon bieneusi* infect the intestines, primarily causing intestinal tissue damage, diarrhea, and weight loss (Tao et al. 2015; Liu et al. 2013).

**TABLE 3:** Parasitic infections in Giant pandas.

Category	Pathogen	Prevalence	Sample type	Detection method	Management status	Sampling sites	Sampling time	References
Protozoa	*Enterocytozoon bieneusi*	8.70% (4/46 samples)	Feces	PCR, sanger sequencing	Captive	Shaanxi Province: Rare Wild Animal Rescue and Breeding Research Center, Qinling Wildlife Park	2013.09–2014.06	Tian et al. (2015)
	*Enterocytozoon bieneusi*	34.5% (69/200 samples)	Feces	PCR, sanger sequencing	Captive	Panda Conservation Bases and Zoos across China	2016.05–2017.06	Li et al. (2018)
	*Cryptosporidium andersoni*	captive: 15.57% (19/122 samples), wild: 0.5% (1/200 samples)	Feces	PCR, sanger sequencing	Captive and wild	Chengdu Panda Breeding Research Base, China Conservation and Research Center for the Giant Panda (wild population), Habitats in the Daxiangling, Liangshan mountain ranges of Sichuan Province	N/A	Tao et al. (2015)
	*Cryptosporidium* sp.	1.75% (1/57 samples)	Feces	Microscopic examination	Captive	Bifengxia Base in Ya'an, Sichuan	2010.12, 2011.03	Liu et al. (2012)
	*Cryptosporidium* sp. (panda genotype)	1.7% (1/57 samples)	Feces	Microscopic examination	Captive	Ya'an City, Sichuan Province, China	2010.11, 2011.03	Liu et al. (2013)
	*Toxoplasma gondii*	35.67% (56/157 giant pandas)	Blood	ELISA, MAT	Captive	Sichuan, Shaanxi, Zhejiang, Taiwan	2007–2022	Yue et al. (2022)
	*Toxoplasma gondii*	100% (1/1 giant panda)	Blood, tissue	PCR, indirect immunofluorescence assay	Captive	Zhengzhou Zoo	2014.02	Ma et al. (2015)
	*Toxoplasma gondii*	37% (7/19 giant pandas)	Blood	Indirect hemagglutination test	Captive	Chengdu Research Base of Giant Panda Breeding	1998–2003	Loeffler et al. (2007)
	*Toxoplasma gondii*	1.4% (1/69 samples)	Blood	Indirect hemagglutination test	Captive and wild	Chengdu Research Base of Giant Panda Breeding	2012–2014	Zhong et al. (2014)
	*Sarcocystis* sp.	2.27% (1/44 samples)	Feces	Microscopic examination	Wild	Foping National Nature Reserve in Shaanxi	2010–2012	Hu et al. (2007)
Nematoda	*Toxascaris seleactis*	1.84% (3/163 samples)	Feces	Microscopic examination	Wild	Foping National Nature Reserve	2013.09–2018.04	Zhou et al. (2020)
	*Strongylida* sp.	0.11% (3/2680 samples)	Feces	Microscopic examination	Wild	28 Counties and 9 Nature Reserves in Sichuan and Gansu	1985–1988	Lai et al. (1993)
	*Baylisascaris schroederi*	88% (44/50 samples)	Feces	PCR, sanger sequencing	Captive	Bifengxia in Ya'an, Sichuan	N/A	Zhou et al. (2013)
	*Baylisascaris schroederi*	68% (34/50 samples)	Feces	PCR, microscopic examination	Captive	Bifengxia Base in Ya'an, Sichuan	N/A	Wang, Li, et al. (2013)
	*Baylisascaris schroederi*	54% (49/91 samples)	Feces	PCR, capillary electrophoresis‐single strand conformation polymorphism	Wild	Tangjiahe National Nature Reserve in Sichuan	2009–2010	Diemert et al. (2012)
	*Baylisascaris schroederi*	34.1% (30/88 samples)	Feces	Microscopic examination	Captive	Chengdu Research Center for Giant Panda Conservation in Dujiangyan, Sichuan	2018.04–2018.10	Yaxian et al. (2023)
	*Baylisascaris schroederi*	7% (1/14 samples)	Feces	Microscopic examination	Captive	Chengdu Zoo	1997–1998	Chengdong et al. (2001)
	*Baylisascaris schroederi*	56.15% (1505/2680 samples)	Feces	Microscopic examination	Wild	28 Counties and 9 Nature Reserves in Sichuan and Gansu	1985–1988	Lai et al. (1993)
	*Baylisascaris schroederi*	96.93% (158/163 samples)	Feces	Microscopic examination	Wild	Foping National Nature Reserve	2013.09–2018.04	Zhou et al. (2020)
	*Baylisascaris schroederi*	52.3% (101/193 samples)	Feces	Microscopic examination	Wild	Foping National Nature Reserve	2012	Peng et al. (2017)
	*Baylisascaris schroederi*	55.17% (48/87 giant pandas)	Feces	Microscopic examination	Wild	Nature Reserves in Sichuan, Gansu, Shaanxi and other regions	N/A	Zhang et al. (2014)
	*Baylisascaris schroederi*	54.0% (68/126 giant pandas)	Feces	Microscopic examination	Wild	Six Mountain Ranges including Qinling	2006–2008	Lei et al. (2011)
	*Baylisascaris schroederi*	27.3% (33/121 samples)	Feces	Microscopic examination	Wild	Foping Nature Reserve	2017.4	Ma et al. (2021)
	*Toxocara ursi*	4.51% (121/2680 samples)	Feces	Microscopic examination	Wild	28 Counties and 9 Nature Reserves in Sichuan and Gansu	1985–1988	Lai et al. (1993)
	*Bunostomum* sp.	4.54% (2/44 samples)	Feces	Microscopic examination	Wild	Foping National Nature Reserve in Shaanxi	2010–2012	Hu et al. (2007)
	*Ancylostoma ailuropodae n*. sp.	1.23% (2/163 samples)	Feces	Microscopic examination	Wild	Foping National Nature Reserve	2013.09–2018.04	Zhou et al. (2020)
	*Metastrongyloidea*	0.04% (1/2680 samples)	Feces	Microscopic examination	Wild	28 Counties and 9 Nature Reserves in Sichuan and Gansu	1985–1988	Lai et al. (1993)
Trematoda	*Ogmocotyle* sp.	6.82% (3/44 samples)	Feces	Microscopic examination	Wild	Foping National Nature Reserve in Shaanxi	2010–2012	Hu et al. (2007)
	*Ogmocotyle* sp.	0.48% (13/2680 samples)	Feces	Microscopic examination	Wild	28 Counties and 9 Nature Reserves in Sichuan and Gansu	1985–1988	Lai et al. (1993)
Ectoparasites	*Dermatophagoides*	100% (7/7 samples)	Skin hair samples	Microscopic examination	Captive	Chengdu Zoo	1997–1998	Chengdong et al. (2001)

*Note:* Column Definitions: Category: Taxonomic family or genus of the parasite; Pathogen: Specific parasite species; Prevalence: Percentage of positive samples or giant pandas relative to the total number of samples or giant pandas tested; Sample Type: Samples used for detection; Detection Method: Primary technique employed for pathogen identification; Management Status: Indication of the host (giant panda) as captive or wild; Sampling Sites: Geographical location or institution where samples were collected; Sampling Time: Year or period when samples were collected; References: Corresponding to the source of the study.

Abbreviations: N/A, not available; PCR, polymerase chain reaction; ELISA, enzyme‐linked immunosorbent assay; MAT, modified agglutination test.

Besides endoparasites, ectoparasite infections are frequently reported and represent the second most prevalent parasitic problem (Li et al. 2020). Ticks (prevalence 2%–100%), *Haemaphysalis ailuropodae* (prevalence 50%), and *Chorioptes panda* causes significant harm. Infections with *Demodex ailuropodae* and *C. panda* cause sarcoptic mange, with prevalence ranging from 66.7% (6/9) to 100% (7/7) (Li et al. 2020). Ticks are the second most important blood‐feeding arthropods after mosquitoes. Thirteen tick species have been identified on wild giant pandas to date, belonging to the genera *Haemaphysalis* (nine species), *Ixodes* (three species), and *Dermacentor* (one species). Mixed tick infestations are common, with *Haemaphysalis flava* being the most prevalent in panda populations (Wang et al. 2018). These ectoparasites cause anemia, dermatitis, and secondary bacterial infections. Tick infestation is characterized by anemia, malnutrition, inflammation, and exhaustion in pandas (Zhang et al. 2010). More importantly, tick‐borne diseases can lead to devastating secondary infections by other pathogens (viruses, bacteria, parasites). Ectoparasites, particularly ticks, are proven vectors for vertebrate pathogens and facilitate interactions and genetic exchange among viruses (Ma et al. 2022). Using viral metagenomics, Rui Ma et al. identified 32 viruses in ticks collected from two giant pandas in the Daxiangling Nature Reserve; half showed homology to viruses carried by pandas and sympatric animals (e.g., red pandas [ *Ailurus fulgens* ]), indicating frequent viral exchange between ticks and their hosts, like pandas (Ma et al. 2022). Several phylogenetically classified viruses (Bunyaviruses, Hepacivirus‐like, Circovirus‐like) were relatively abundant, though whether these novel tick‐associated viruses can replicate in ticks and be transmitted to host animals during blood‐feeding requires further study. These findings expand our understanding of the role of panda‐associated ticks in local ecosystems, particularly in virus acquisition and transmission, and lay the groundwork for assessing panda exposure risks to tick‐borne viruses, reporting numerous cases of viral host‐switching among sympatric species. Regarding bacteria, approximately 10% of all tick species (over 900) play significant roles in transmitting agents of diseases like borreliosis and rickettsiosis (Ma et al. 2022). However, detailed studies on tick‐borne bacterial diseases within panda reserves are currently lacking. While a direct causal link between tick‐borne pathogens and mammalian disease in pandas cannot be definitively established, it is evident that many viruses carried by ticks are genetically related to viruses found in pandas and other hosts sharing adjacent habitats (Ma et al. 2022). *Babesia* spp., tick‐borne protozoan blood parasites, can cause hemolytic anemia, thrombocytopenia, lethargy, and splenomegaly in pandas (Ma et al. 2024). Ticks serve as vectors for *Babesia*, and babesiosis is primarily transmitted between hosts via tick bites in natural environments (Tsai et al. 2024). Other parasites may act as opportunistic pathogens, causing disease or exacerbating conditions when immunity is compromised by viral or bacterial infections (Ma et al. 2024).

Regular deworming and environmental disinfection can effectively protect captive pandas from parasitic diseases. However, frequent drug overuse in captive settings has led to the development of drug resistance in parasites, particularly against common anti‐ *B. schroederi* anthelmintic. Furthermore, drug resistance genes carried by captive pandas can be disseminated through reintroduction programs (Li et al. 2020). Tao Wang et al. (Wang et al. 2018) found high levels of gene flow for *B. schroederi* among panda populations in different habitats, indicating the potential for rapid spread of anthelmintic resistance, posing a major obstacle to controlling this parasite and necessitating integrated management approaches. Developing sensitive, convenient, and high‐throughput detection methods for panda parasites is another crucial aspect for assessing prevalence and distribution in captive and wild populations (Wang et al. 2018). Critically, the infectivity, pathogenicity, range, prevalence, and vectors for parasites in wild populations remain largely unknown. Further research is needed to elucidate pathogen–host–vector relationships and determine the necessity for targeted conservation management actions for this vulnerable species.

### 2.4 Multidimensional Drivers of Variations in Pathogen Infection Rates Among Giant Pandas

In wildlife epidemiological studies, identifying the factors contributing to variations in pathogen prevalence is particularly crucial for formulating conservation strategies for rare and endangered species. As a flagship species in global biodiversity conservation, the pathogen infection status of giant pandas has attracted considerable attention. However, substantial discrepancies exist in infection rate data reported across different studies (Tables 1, 2, 3), posing challenges for disease risk assessment. Through a comprehensive synthesis analysis, this review reveals that these discrepancies are not random but are closely associated with multiple factors, including the temporal span of investigation, seasonal selection, geographical scope, giant pandas' management status (wild/captive), and sample types. Based on specific survey data (Tables 1, 2, 3), this section systematically dissects the impacts of these five key factors on the variations in pathogen prevalence in giant pandas.

Firstly, under consistent conditions regarding detection methods, survey locations, and others, temporal factors exert a critical influence on driving differences in pathogen prevalence in giant pandas. Investigations conducted at the Chengdu Research Base of Giant Panda Breeding using the indirect hemagglutination assay showed that the infection rate of *T. gondii* decreased from 37.0% (7/19) during 1998–2003 (Loeffler et al. 2007) to 1.4% (1/69) during 2012–2014 (Zhong et al. 2014). Similarly, microscopic examination of fecal samples in the Foping National Nature Reserve revealed that the infection rate of *B. schroederi* declined from 52.3% (101/193) in 2012 (Peng et al. 2017) to 27.3% (33/121) in 2017 (Ma et al. 2021). The temporal variation in infection rates may be related to changes in ecological conditions and management practices. Enhanced conservation and management measures, population dynamics shifts, and changes in external environmental climates likely have contributed to reduced infection risks of certain pathogens in recent years. Long‐term longitudinal monitoring is essential in wildlife epidemiological research, as it can reveal dynamic trends and driving factors of pathogen prevalence, thereby providing a scientific basis for evaluating the effectiveness of prevention and control measures and formulating adaptive management strategies.

Secondly, seasonality, mediated through environmental factors such as temperature, humidity, and solar radiation, along with the activity cycles of vectors and other animals, collectively shapes the transmission dynamics of pathogens and represents another critical factor influencing variations in pathogen prevalence in giant pandas. For instance, in studies on *B. schroederi* infection in the Foping National Nature Reserve, a survey covering all seasons from September 2013 to April 2018 reported an infection rate of 96.93% (158/163) (Zhou et al. 2020), whereas a survey conducted only in April (spring) 2017 showed a markedly lower rate of 27.3% (33/121) (Ma et al. 2021). Additionally, regarding the detection of *Klebsiella pneumoniae* , one study conducted in spring, summer, and autumn reported a carriage rate of 59.02% (90/153) (Feng et al. 2022), while another year‐round study showed an overall carriage rate of 23% (42/182) (Yan et al. 2022). For parasitic pathogens, their life cycles, host exposure risks, and infection intensities often exhibit seasonal fluctuations corresponding to climate and host behaviors, with spring potentially representing a period of lower infection intensity. For bacterial pathogens, warmer seasons typically favor their survival and reproduction, and host physiological activities are also more active, leading to higher detection rates. Conversely, low winter temperatures may inhibit the persistence of bacteria in the external environment, thereby reducing exposure risks. Therefore, systematic surveillance across multiple seasons and time periods is necessary to accurately characterize seasonal fluctuation patterns of different pathogens, identify high‐risk seasons, and develop targeted prevention and control strategies.

Thirdly, the geographic scope of a study represents another important factor contributing to variations in giant pandas' pathogen infection rates. For example, the infection rate of *Enterocytozoon bieneusi* was reported as 8.70% (4/46) within Shaanxi Province (Tian et al. 2015), whereas surveys across multiple panda conservation bases and zoos nationwide reported a higher rate of 34.5% (69/200 samples) (Li et al. 2018). Similarly, the infection rate of *Ogmocotyle* sp. was 6.82% (3/44) in wild giant pandas in the Foping Nature Reserve of Shaanxi (Hu et al. 2007), but only 0.48% (13/2680) in a broader survey covering 28 counties and cities in Sichuan and Gansu Provinces (Lai et al. 1993). For canine parvovirus, the infection rate was 90% (40/44) at the Chengdu Research Base of Giant Panda Breeding (Loeffler et al. 2007), compared with 58% (54/92) in surveys conducted in multiple locations, including Sichuan and Beijing (Qin et al. 2010). Such discrepancies related to the survey scale may be attributed to specific environmental and management factors. Infection rates from localized surveys only reflect the conditions within specific subpopulations, while broader multi‐regional sampling covers diverse geographical and ecological conditions, providing a more accurate representation of the overall infection level of a specific pathogen in the giant panda species. Therefore, epidemiological assessments of flagship species should explicitly consider spatial scale.

Fourthly, the pathogen profile in giant pandas is closely linked to their management status (wild/captive). Taking *Cryptosporidium andersoni* as an example, studies using PCR combined with sequencing reported an infection rate of 15.57% (19/122) in captive populations at the Chengdu Research Base (Tao et al. 2015), compared with only 0.5% (1/200) in wild pandas from mountain ranges such as Daxiangling and Liangshan in Sichuan (Tao et al. 2015) Province. A similar trend was observed in serological testing for canine distemper virus: the infection rate was 0 (0/8) in wild giant pandas in Foping (Susan et al. 1994) but it reached 84% (37/44) in captive populations in Chengdu (Loeffler et al. 2007). The underlying reasons may include higher population density, frequent individual contact, and close interaction with humans and other animals in captive settings, all of which can facilitate the transmission of certain pathogens. In contrast, the low‐density population distribution, extensive activity spaces, and reduced exposure to potential infection sources in the wild generally lower infection risks for these pathogens. Consequently, disease prevention and control strategies for giant pandas should be differentiated: for captive populations, emphasis should be placed on strengthening biosecurity management in husbandry, implementing regular strict quarantine, vaccination, and health monitoring; for wild populations, efforts should focus on habitat conservation, preventing spillover of zoonotic diseases, and when necessary, conducting non‐invasive monitoring and ecological interventions. Only by recognizing the epidemiological differences between wild and captive populations can comprehensive and effective conservation plans be formulated. Furthermore, current research on pathogens in giant pandas exhibits a notable sample bias. Among the 52 studies included for analysis, 75.0% (39 studies) involved only captive individuals, 19.2% (10 studies) focused solely on wild populations, and merely 5.8% (three studies) encompassed both (Tables 1, 2, 3). This bias largely stems from systematic difficulties in obtaining wild samples, including remote habitats, low population densities, and ethical constraints (Deem et al. 2001). This pronounced captive‐oriented bias results in existing pathogen data—particularly records of viruses and bacteria—being predominantly derived from captive or rescued individuals. These individuals differ systematically from wild populations in terms of health status, diet, antibiotic exposure, and population density (Xue et al. 2015). Consequently, data obtained from captive individuals cannot be directly extrapolated to wild populations and may inaccurately reflect the true prevalence levels, antimicrobial resistance profiles, or pathogen composition within wild herds, thereby compromising the reliability of disease risk assessments (Zhao, Li, et al. 2023). This limitation not only hinders the accurate evaluation of pathogen ecology and disease risks in natural ecosystems but also constrains our understanding of the differences between wild and captive populations. Therefore, to enhance data representativeness and the accuracy of epidemiological inferences, it is imperative to collaborate across institutions to establish standardized, long‐term health monitoring sites in key habitats. This will facilitate systematic long‐term field surveillance and comparative studies across populations.

Finally, the sample type used also directly affects the detection rate of pathogens in giant pandas. For instance, in the detection of CDV, the infection rate was 80% (4/5) using fecal and blood samples (Zhao et al. 2017), whereas it was only 27% (6/22) with nasal swabs and tissue samples (Feng et al. 2016). Similarly, for *P. mirabilis* , the detection rate was 4.2% (1/24) using vaginal swabs (Yang et al. 2016), but reached 11.5% (17/148) in fecal samples (Yizhou et al. 2025). These discrepancies are likely attributed to differences in the inherent characteristics of the samples and the scope of detection for specific pathogens. Fecal and blood samples often contain higher or more sustained viral loads during infection, making them more conducive to detection. In contrast, nasal swabs and tissue samples are more susceptible to factors such as infection stage and sampling timing, leading to a higher risk of false negatives. For *P. mirabilis* , the lower detection rate with vaginal swabs may reflect the local colonization status of the reproductive tract, where natural colonization is typically low. Fecal samples, on the other hand, provide a more comprehensive picture of intestinal colonization and can be obtained non‐invasively, making them more suitable for population‐level screening. Therefore, in epidemiological surveys of giant panda pathogens, it is essential to scientifically select sample types based on the biological characteristics and transmission patterns of the target pathogens to accurately assess the prevalence status and provide reliable evidence for health management.

## 3 Pathogen Carriage by Domestic and Wild Animals in Giant Panda Nature Reserves

Numerous pathogens are transmitted to giant pandas through direct or indirect contact with free‐roaming domestic animals (e.g., livestock, stray dogs and cats) and sympatric wildlife within nature reserves. These interactions create complex transmission networks and potential risks of cross‐species disease outbreaks. Therefore, a comprehensive understanding of the spatial distribution of domestic animals, wildlife, and their associated pathogens is fundamental for assessing the risks and mechanisms of infectious disease transmission in giant panda nature reserves (Table S2).

### 3.1 Domestic Pets

Domestic dogs and cats, especially those free‐roaming in or near giant panda reserves, represent significant sources of infectious agents. Their relatively high population densities, frequent contact with humans, and poor immunization increase the risk of introducing zoonotic and multi‐host pathogens into wildlife populations. Dogs are implicated in the extinction of 11 species and pose threats to over 188 others (Doherty et al. 2017). Panda reserves are often closely linked to human settlements, allowing dogs to roam freely over distances exceeding 10 km overnight; some feral dogs even establish permanent residence within reserves. A comprehensive survey in six village groups surrounding Liziping Reserve found 64% (212/334) of dogs were free‐roaming (Callan et al. 2020). Across all panda reserves, 40% of panda habitat falls within the roaming range of stray dogs. Between 2013 and 2014, dog densities in eight counties surrounding Foping National Nature Reserve (Zhouzhi, Hu, Ningshan, Foping, Yang, Chenggu, Liuba, Taibai) ranged from 3.71 to 20.80 dogs/km^2^, densities sufficient to sustain transmission of canine infectious pathogens (Yipeng et al. 2017). Since dogs and pandas may share habitats or water sources, the potential for direct or indirect contact between the two species increases, elevating disease transmission risks (Fang et al. 2024). Canine distemper virus (CDV), a known cause of mortality in pandas, has been transmitted from domestic dogs (Yipeng et al. 2017). In Liziping Reserve, 21% of surveyed village dogs tested seropositive for antibodies against at least one of four viruses (CDV, CPV, Rotavirus, Rabies virus) known to be fatal to pandas, and many terrestrial mammals within the reserve (e.g., red pandas, yellow‐throated martens [ *Martes flavigula* ]) are susceptible (Liu et al. 2022). Beyond viruses, 67% of dog fecal samples were infected with at least one gastrointestinal parasite; hookworms (*Ancylostoma caninum*) and threadworms (*Strongyloides* sp.) found in dogs can also infect pandas. Dogs provide pathways for disease transmission into wildlife populations, potentially causing epidemics or extinctions across multiple species (McCarthy et al. 2007).

### 3.2 Livestock

Field investigations reveal that cattle, sheep, and horses frequently roam freely into protected areas. Livestock grazing has become the second most common anthropogenic disturbance to panda reserves (after logging), affecting 19% of the reserve area in the Minshan Mountains (Hull et al. 2014). Livestock numbers have increased significantly in some areas over recent years; for instance, livestock numbers in Wanglang National Nature Reserve increased nine‐fold over 15 years (Wang, Lan, et al. 2022). Pathogen transmission between livestock and wildlife at these shared interfaces is often bidirectional. Approximately 77% of livestock pathogens are capable of infecting multiple host species, including wildlife (Hull et al. 2014; Kang et al. 2020). In panda habitats, livestock feces may contaminate water and food sources, substantially raising the risk of pathogen ingestion by pandas, who spend over 14 h a day feeding. Therefore, the spillover of livestock pathogens to pandas and other wild species poses substantial conservation challenges. Despite these concerns, systematic surveillance of pathogens shared by livestock and wildlife across the network of panda reserves remains insufficient.

### 3.3 Wild Sympatric Species

Panda nature reserves also serve as sanctuaries for numerous sympatric endangered and vulnerable species. Species benefiting from habitat protection span six orders and 18 families, including one bird order (Galliformes) and five mammal orders: Primates (e.g., Sichuan snub‐nosed monkey [ *Rhinopithecus roxellana* ], Proboscis monkey [ *Nasalis larvatus* ], Tibetan macaque [ *Macaca thibetana* ]), Carnivora (e.g., Asian black bear [ *Ursus thibetanus* ], Gray wolf [ *Canis lupus* ], Red fox [ *Vulpes vulpes* ], red pandas, Hog badger [ *Arctonyx collaris* ], Masked palm civet [ *Paguma larvata* ], Asian golden cat [ *Catopuma temminckii* ], leopard cat [ *Prionailurus bengalensis* ]), Artiodactyla (e.g., Tufted deer [ *Elaphodus cephalophus*], Sambar deer [ *Rusa unicolor* ], Chinese muntjac [ *Muntiacus reevesi* ], Takin [ *Budorcas taxicolor* ], Chinese serow [ *Capricornis milneedwardsii* ], Chinese goral [ *Naemorhedus griseus* ], wild boar), Rodentia (e.g., Chinese bamboo rat [ *Rhizomys sinensis* ], Malayan porcupine [ *Hystrix brachyura* ], Himalayan marmot [ *Marmota himalayana* ]), and Lagomorpha (e.g., Gansu pika [ *Ochotona cansus* ], Plateau pika [ *Ochotona curzoniae* ]; Tang et al. 2022; Liu, Shen, et al. 2023). This sympatric wildlife shares ecological space and resources with giant pandas, which may facilitate pathogen transmission. However, there has been no systematic investigation or research on the pathogens carried by these animals and their potential impacts. Through non‐invasive sampling, high‐throughput sequencing analyses targeting viruses, bacteria, and parasites are imperative for establishing a pathogen repository of sympatric species. This is crucial for investigating pathogens within protected areas and characterizing their distribution patterns and pathogenic traits. It is therefore recommended to establish a “sympatric species pathogen inventory program” and enhance monitoring feasibility through non‐invasive sampling methods such as fecal or skin shedding collection.

## 4 Cross‐Species Transmission Mechanisms of Pathogens in Giant Panda Reserves

Pathogens can be transmitted among giant pandas and between pandas and other species through various direct and indirect mechanisms. For example, the roundworm ( *B. schroederi* ) can infect pandas primarily via fecal–oral transmission: (1) eggs adhere to the feet of pandas after they walk across contaminated ground and are subsequently ingested when pandas handle bamboo with their forepaws; (2) pandas mark trees using urine or feces, and parasites can be transmitted when another panda rubs its nose or licks the same site. Increased panda density can significantly elevate the risk of such fecal–oral transmission (Zhang et al. 2008). The high diversity of sympatric wildlife species in panda habitats, and their frequent direct or indirect contact, creates favorable conditions for interspecies and cross‐species pathogen transmission. Intermediate hosts increase the likelihood of cross‐species transmission, potentially driving viral adaptation and evolution, enhancing pathogenicity and transmissibility, and posing significant threats (Yipeng et al. 2017; Fang et al. 2024). For example, several animal groups (camelids, mustelids, canids, procyonids, felids, ursids, viverrids, non‐human primates) are highly susceptible to CDV and can act as hosts, facilitating virus transmission between dogs and pandas (Feng et al. 2016; Wilkes 2022). Stray cats or rodents infected with *T. gondii* could be sources of fatal infections in pandas (Ma et al. 2015). Additionally, vectors like ticks play a crucial role in disease transmission by exchanging pathogens with vertebrate hosts during blood‐feeding. Studies show that ticks infesting pandas harbor broad viral genetic diversity and share numerous viruses with pandas (Ma et al. 2022).

**FIGURE 2:**
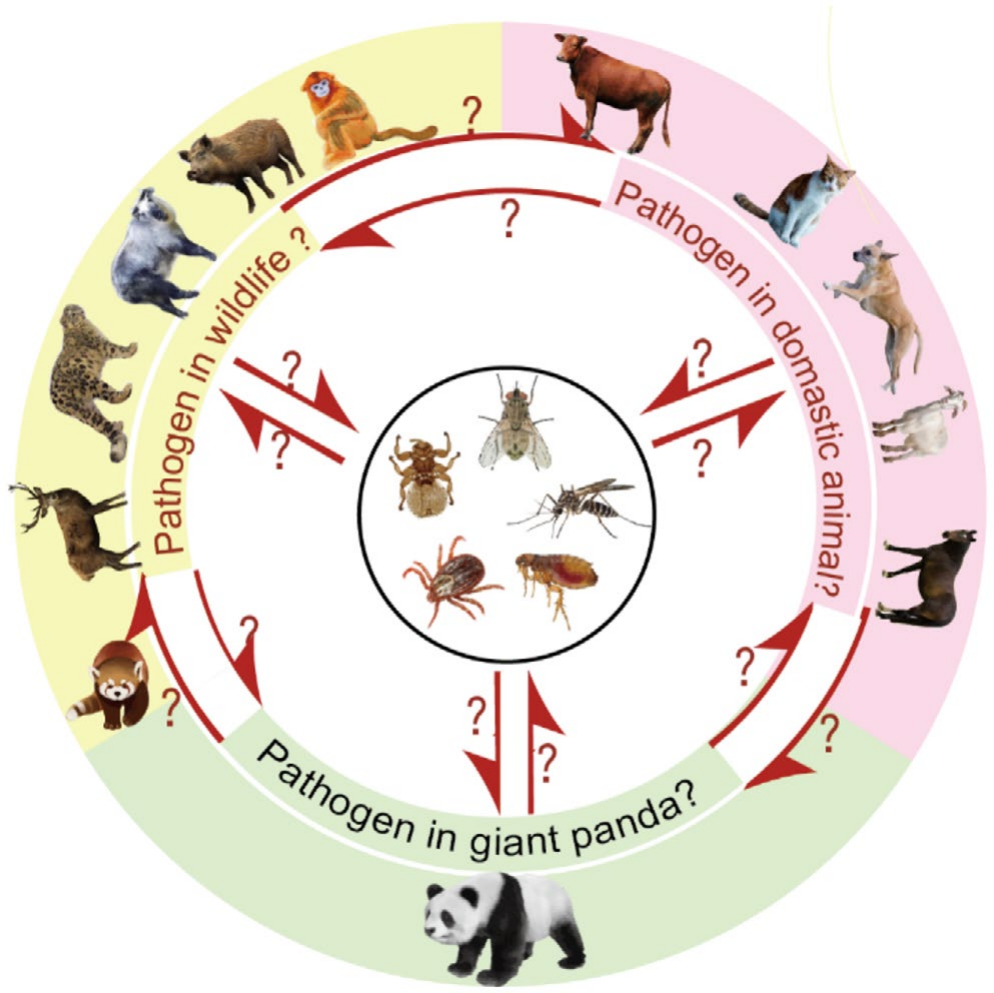
Pathways of multi‐host pathogen transmission among giant pandas, sympatric wildlife, and domestic animals in nature reserves.

The multi‐host nature and host adaptability of pathogens increase the likelihood of cross‐species transmission and complicate control efforts. The rich diversity of species within panda reserves and their direct or indirect contacts create favorable conditions for pathogen spread. Carnivore protoparvovirus 1, represented by FPV and CPV, is globally distributed with a broad host range. FPV and CPV infections have been documented in diverse species, including raccoons, lions, leopards, giant pandas, and tigers ( *Panthera tigris* ; Zhao, Hu, et al. 2023). *Hepatozoon* sp. are apicomplexan blood parasites capable of infecting various vertebrates globally, including canids, ursids, felids, and many others (Yu et al. 2019). Other multi‐host pathogens include Rotavirus, Rabies Virus, *Hepatozoon* sp., and *T. gondii*. Given the close contact among pandas, domestic animals, and sympatric wildlife, it is crucial to investigate the transmission dynamics and genetic diversity of pathogens across hosts. FPV strains detected in fecal samples from stray cats within reserves showed > 99% similarity in coding regions to FPV found in giant pandas and red pandas (Min et al. 2022). This high genetic relatedness suggests the possibility of cross‐species transmission among “giant panda, red panda and cat”. Due to rapid evolution and transmission, the host range of some viruses, like parvoviruses and CDV, continues to expand, threatening multiple endangered wildlife species (Zhao, Hu, et al. 2023). Furthermore, beyond known viruses, a metaviromic survey of giant pandas and sympatric animals and vectors (red pandas, stray cats, wild rodents, mosquitoes) within the same habitat identified 250 viral genomes, revealing highly diverse viral communities across multiple animal species in the ecosystem and emphasizing the need to study the prevalence, risk, and significance of carnivore infectious diseases in pandas (Min et al. 2022). These studies demonstrate potential cross‐species transmission events of pathogens between pandas and sympatric animals within the same ecosystem, revealing the genetic diversity and transmission relationships of different viral pathogens within the “panda‐sympatric wildlife‐arthropod‐domestic animal” nexus. However, several critical aspects regarding pathogens associated with diverse animal hosts, including their transmission dynamics, host range, pathogenic mechanisms, and disease manifestations, require further elucidation (Figure 2).

It is crucial to emphasize that the currently prevailing reactive disease management model has become a major impediment to achieving breakthroughs in pathogen prevention and control for giant pandas. At present, disease management in giant pandas predominantly involves pathogen identification and localized containment only after disease outbreaks or individual deaths occur. Furthermore, there is a persistent lack of a systematic, proactive surveillance network grounded in eco‐epidemiological principles. This model has resulted in long‐term uncertainty regarding the pathogen reservoir, characteristics, and transmission dynamics, directly undermining the proactivity and effectiveness of prevention efforts and leading to multiple hazardous incidents. Since 1986, fatal cases of hemorrhagic enteritis in giant pandas have occurred frequently in Chengdu and Wolong, with mortality rates among young individuals once reaching 100%. The absence of prior baseline pathogen surveys delayed for years the identification of *enteroinvasive Escherichia coli* as the causative agent, thereby missing the critical window for early intervention (Li et al. 1995). In captive populations, the detection rates of hepatitis E virus (HEV) in liver and kidney samples from deceased individuals were as high as 88.9% and 85.7%, respectively, whereas no related antigens were detected in surviving individuals. This finding underscores the importance of routine individual monitoring and understanding pathogen traits, such as latency periods, for epidemic control (Zhang et al. 2013). In 2020, following the death of a wild juvenile giant panda in Wolong due to infection with canine parvovirus type 2c (CPV‐2c), subsequent investigations revealed the presence of the virus in fecal samples from various wild animals within the reserve, including giant pandas and leopard cats. This case highlights how insufficient knowledge of the viral host range, transmission routes, and cross‐species transmission risks hindered the timely recognition of epidemic spread (Yang et al. 2022). These cases collectively demonstrate that a reactive response model fails to facilitate timely diagnosis and tracking of pathogen transmission, ultimately leading to outbreaks. Therefore, establishing a prospective, proactive surveillance network—through systematic baseline pathogen surveys, tracking of pathogen ecological traits, and monitoring of cross‐species transmission pathways—enables the early identification of epidemic risks and timely warning interventions. This shift is fundamental to transforming giant panda disease prevention and control from a post‐outbreak response model to one capable of pre‐emptive risk assessment.

To achieve timely and effective surveillance of the complex pathogen transmission networks described above, it is essential to integrate pathogen molecular data with animal ecological data. However, current research tends to separate pathogen molecular studies from animal ecology, often focusing only on the surveillance of specific pathogens or particular animal species. This represents a significant gap in the prevention and control of diseases in giant panda reserves. Systematically understanding the host range, genetic variations, pathogenicity, and other characteristics of pathogens—combined with knowledge of the habitat dynamics of giant pandas, sympatric wildlife, and domestic animals across different temporal and spatial scales—is crucial for assessing the potential of various pathogens to infect, cause disease, and spread among different animal hosts. Such an integrated approach is fundamental to elucidating pathogen transmission mechanisms, enabling early warning of outbreaks in protected areas, and formulating targeted conservation strategies.

In the context of pathogen detection methods, reliance on a single detection technique is insufficient for comprehensive prevention and control. There is an urgent need to establish a surveillance system that integrates traditional methods with modern sequencing technologies. Data presented in Tables 1, 2, 3 indicate that over half of pathogen investigations in giant pandas still depend on traditional techniques such as microscopic observation, serum neutralization tests, and pathogen isolation and culture. While these methods are cost‐effective, technically mature, and yield intuitive results, their detection sensitivity is limited compared to modern molecular techniques. For instance, the detection rates for *Proteus mirabilis* and *Klebsiella pneumoniae* using isolation and culture methods (25%, 83%) were lower than those achieved by multiplex PCR (30%, 90%; Liu, Zheng, et al. 2023); nevertheless, traditional methods can still serve to verify molecular detection results. In another study, the combined use of morphological characteristics, sequence similarity, and phylogenetic analysis led to the first detection of *Babesia* infection (*Babesia* sp. strain EBP0; Yue et al. 2020) in giant pandas. Modern technologies overcome the limitations of traditional methods, which are often laborious, low‐throughput, and restricted to known pathogens. Conversely, traditional methods compensate for the drawbacks of modern techniques, such as high cost, complex data analysis, and limited sensitivity for detecting low‐abundance pathogens. Therefore, constructing a multidimensional surveillance system based on “preliminary screening by traditional techniques combined with precise identification and in‐depth analysis by modern sequencing technologies” and integrating the One Health concept with data on habitat environment and sympatric animals (Gardy and Loman 2017) will elucidate the distribution patterns and evolutionary trends of pathogens in giant pandas. This approach will provide a crucial bioscientific basis for targeted conservation efforts.

## 5 Conclusions and Future Directions

The effectiveness of nature reserves depends on our understanding and management of the complex interactions among humans, domestic animals, and the wildlife we aim to protect, such as the giant panda. Conservation efforts for giant pandas are now shifting from passive, in situ protection toward more proactive and precise intervention strategies. In the foreseeable future, the conservation status of giant pandas will, to a large extent, depend on the advancement of pathogen surveillance and disease prevention measures. Although considerable progress has been made in understanding animal diseases over the past five decades, our current knowledge of disease processes, especially those occurring at the interface of domestic and wild animals, remains limited. Future efforts should focus on the following aspects.

### 5.1 Quantifying Epidemiological Parameters

The lack of accurate quantitative data describing the spatial distribution of giant panda sympatric animals constrains our understanding of pathogen transmission. Therefore, future efforts need to collect essential data for describing disease risks involving humans, domestic animals, and wildlife, including animal species distribution, population sizes and structure, and contact rates between and within different animal species.

### 5.2 Establishing a Pathogen and Disease Database

Incomplete information on animal pathogens and disease status within reserves, a narrow focus on a limited number of known viruses (e.g., CDV, CPV, influenza A), often targeting specific species rather than the broader host–pathogen network, and a growing number of diseases reported in captive rather than wild pandas limit pathogen research. Therefore, systematically collect samples from livestock (horses, cattle, sheep), domestic pets (dogs, cats), giant pandas, various sympatric wildlife, and vectors (ticks, mosquitoes) at the wildlife–domestic animal interface within panda reserves. Utilize metagenomics and metaviromics sequencing and analysis to establish a wildlife pathogen community database for early pathogen discovery and characterization.

### 5.3 Pathogen Characterization and Monitoring

Analyze pathogen prevalence (occurrence and infection intensity), host range, cross‐species transmission potential, pathogenicity, and resistance profiles. Elucidate the diversity and spatiotemporal distribution of pathogens carried by pandas and sympatric wildlife, along with factors influencing cross‐species transmission. Establish robust surveillance systems.

### 5.4 Integrative Analysis of Molecular and Ecological Data

Systematically collected ecological data from stray dogs and cats, domestic animals, tick vectors, sympatric wildlife, and giant pandas are correlated. Through high‐throughput sequencing and genomic epidemiology approaches, pathogen lineages shared across hosts are identified, and their antimicrobial resistance and virulence profiles are characterized. By synthetically interpreting host interactions and spatiotemporal pathogen dynamics, potential transmission routes and directions are reconstructed, thereby elucidating the transmission structure of “who infected whom, when, and where.” This deep integration of molecular and ecological data transforms discrete pathogen detection outcomes into predictive insights regarding pathogen transmission dynamics within the ecosystem. It supports precise, proactive disease prevention and control strategies, achieving a critical leap from characterizing pathogen traits to assessing real‐world exposure risks.

### 5.5 Risk Assessment and Integrated Management

Unravel the bidirectional transmission potential of key pathogens between pandas and domestic animals. Assess spillover/spillback risks. Systematically identify the sources and intensity of disease threats to pandas and domestic animals. Integrate wildlife pathogen surveillance with livestock and human surveillance systems. Develop innovative technologies for enhanced pathogen identification. Utilize identified diseases to guide risk assessment and prioritize investments. Design and implement robust, open‐access early warning systems. Improve understanding of current/recent outbreaks. Share information/data and build pathogen surveillance capacity, particularly in wildlife. Address risks from pandemics/epidemics, increased disease risk and susceptibility, and vector‐borne disease expansion. Promote early intervention by mitigating the lack of pathogen early warning and surveillance methods. Provide scientific and technological support for wildlife disease prevention and control, species conservation, and biodiversity maintenance.

**FIGURE 3:**
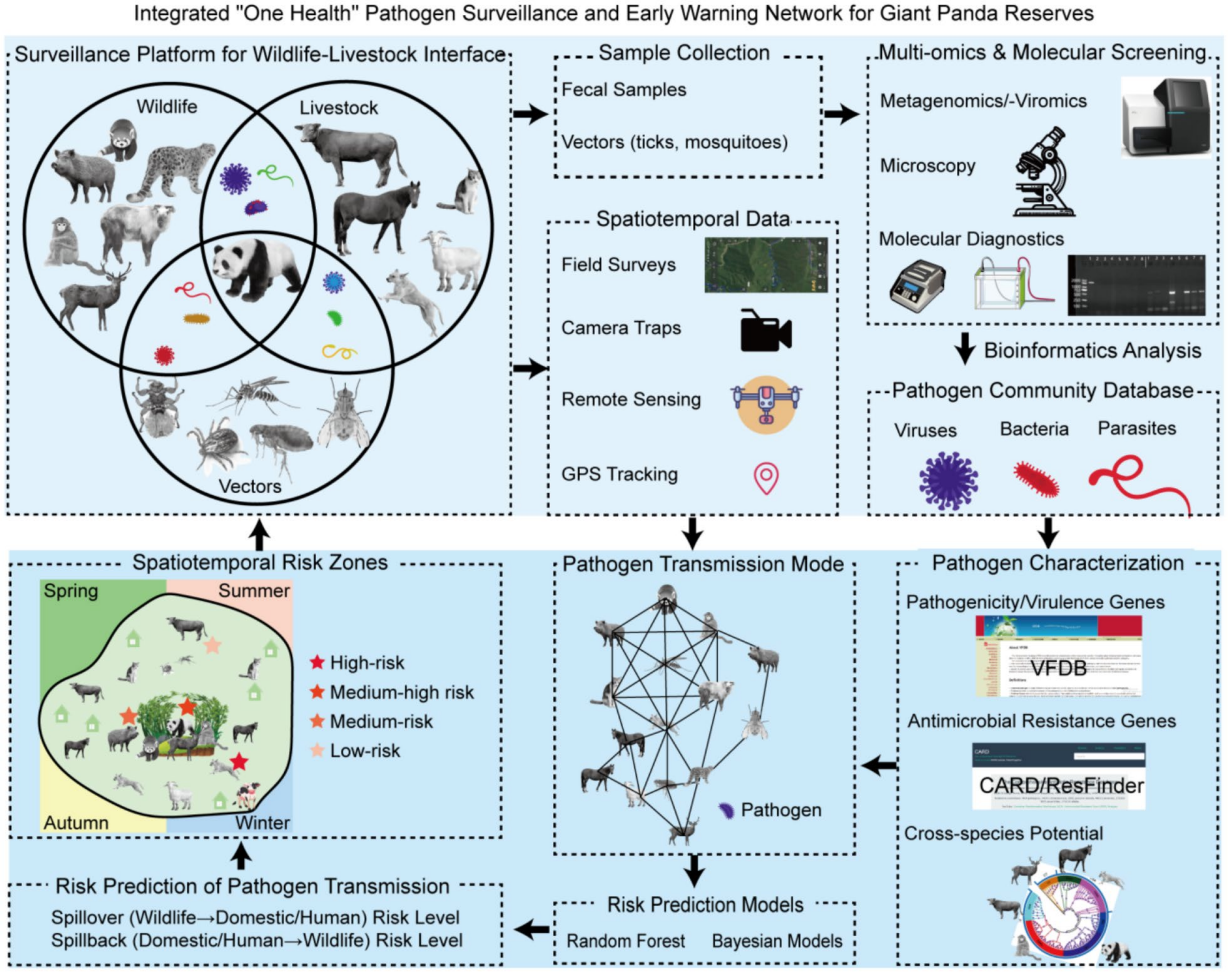
Integrated “One Health” Pathogen Surveillance and Early Warning Network for Giant Panda Reserves.

To effectively manage pathogens within panda reserves, it is crucial to establish targeted early warning systems and surveillance networks at the wildlife–livestock interface for emerging risk identification. Here, we propose a Workflow for an Early Warning System (Figure 3) for Diseases in Giant Pandas and Sympatric Wildlife/Domestic Animals. (1) Pathogen Baseline Survey. Collect fecal samples and vectors (ticks, mosquitoes, fleas) from giant pandas, sympatric wildlife (e.g., wild boar, Sichuan snub‐nosed monkey, Takin, Leopard cat), and domestic animals (cattle, sheep, horses, dogs, cats). Employ metagenomics/viromics, microscopy, and molecular diagnostics for systematic pathogen screening at the wildlife–domestic animal interface. Utilize bioinformatics to identify viruses, bacteria, and parasites, establishing a reserve pathogen database and characterizing pathogen diversity, including key pathogens and zoonoses. Link pathogen data to individual animal information and spatiotemporal data (field surveys, camera traps, remote sensing, GPS tracking) to analyze distribution patterns and inter‐population transmission. (2) Pathogen Characterization & Transmission. Analyze key pathogens (e.g., Canine Distemper Virus, antibiotic‐resistant *E. coli* , hookworms) for pathogenicity (using VFDB for virulence genes), antimicrobial resistance (AMR; using CARD/ResFinder for resistance genes), cross‐species potential, and transmission modes. Assess strain similarity, gene flow/recombination, and host receptor compatibility to identify critical genes for adaptation and predict transmission trends. Map transmission chains and construct host–pathogen interaction networks based on prevalence, vectors, and potential hosts. (3) Transmission Risk Assessment & Early Warning. Elucidate bidirectional pathogen transmission between hosts using interaction networks. Develop risk scoring criteria integrating pathogen factors (prevalence, pathogenicity, AMR, transmissibility) and ecological factors (host/vector diversity/density). Build predictive models (e.g., Random Forest, Bayesian) to assess spillover (wildlife‐to‐domestic/human) and spillback (domestic/human‐to‐wildlife) risk levels. Systematically identify disease threat sources and intensities to establish a Giant Panda National Park Early Warning System. Identify high‐risk spatiotemporal zones for targeted surveillance and control strategies.

## Author Contributions

**Xiaoli Sun:** writing – original draft (equal), writing – review and editing (equal). **Yi Peng:** writing – original draft (equal), writing – review and editing (equal). **Xiaoye Hao:** data curation (equal). **Rong Dong:** writing – review and editing (equal). **Zhilin Wang:** writing – review and editing (equal). **Le Wang:** validation (equal). **Chengdong Wang:** supervision (equal), writing – review and editing (equal). **Xiangdong Wu:** writing – review and editing (equal). **Zheng Chen:** writing – review and editing (equal). **Wenbo Zhang:** writing – review and editing (equal). **Xiaoli Tang:** writing – original draft (equal), writing – review and editing (equal).

## Funding

This study is supported by the National Natural Science Foundation of China (32570606 and 32322015), Study on Key Technologies for Conservation of Wild Giant Panda Populations and Its Habitats within Giant Panda National Park System (CGF2024001).

## Ethics Statement

The authors have nothing to report.

## Consent

The authors have nothing to report.

## Conflicts of Interest

The authors declare no conflicts of interest.

## Supporting information


**Data S1:** ece373260‐sup‐0001‐Supinfo.docx.

## Data Availability

The authors have nothing to report.
