# Supplementary material for: Safeguarding a Flagship Species: Integrated Surveillance of Cross‐Species Pathogen Transmission in Giant Panda Ecosystems
Source: Ecol Evol. 2026 Mar 14;16(3):e73260. doi: 10.1002/ece3.73260 (PMC13093829; doi:10.1002/ece3.73260)
Supplement: Supplementary file 1 — Data S1: ece373260‐sup‐0001‐Supinfo.docx. [file ECE3-16-e73260-s001.docx]

**Supplementary Table 1**: Other Reports of Parasitic, Bacterial and Pathogenic Infections in Giant Pandas

| Pathogen type | Category | Pathogen | Infection Site | Symptoms | Reference |
| --- | --- | --- | --- | --- | --- |
| Virus | Protoparvovirus | Feline panleukopenia virus(FPV) | Intestine | Diarrhea, vomiting, and lethality | (1) |
|  |  |  |  | Vomiting and mild diarrhea | (2) |
|  |  |  |  | Mild diarrhea | (3) |
|  | Coronavirus | Canine Coronavirus(CCV) | Respiratory tract, digestive tract | Fever, diarrhea | (4) |
|  | Morbillivirus | Canine Distemper Virus(CDV) | N/A | N/A | (5) |
|  |  |  |  | Fever | (6) |
|  |  |  |  | N/A | (7) |
| Bacterium | *Staphylococcus* | *Staphylococcus* sp*.* | Intestinal tract |  | (8) |
|  |  |  | Skin |  | (9) |
|  |  | *Staphylococcus xylosus* | N/A |  | (10) |
|  | *Bacteria* | *Campylobacter jejuni* | Intestine (intestinal disease mentioned) |  | (11) |
|  |  | *Arizona bacillus* |  |  |  |
|  | *Escherichia* | *Escherichia coli* | Lungs, spleen, mesenteric lymph nodes |  | (12) |
|  |  |  | Intestinal tract |  | (8, 10, 13-15) |
|  | *Neisseria* | *Neisseria* sp. | Oral cavity |  | (16) |
|  | *Enterococcus* | *Enterococcus* sp*.* | Intestinal tract |  | (8) |
|  |  | *Enterococcus faecium* | Oral cavity |  | (17) |
|  |  | *Enterococcus faecalis* | Lungs, mesenteric lymph nodes |  | (12) |
|  |  |  | Oral cavity |  | (17) |
|  | *Acinetobacter* | *Acinetobacter* sp*.* | Intestinal tract |  | (9) |
|  |  |  | Skin |  | (12) |
|  | *Propionibacterium* | *Propionibacterium* sp*.* |  |  | (15) |
|  | *Pseudomonas* | *Pseudomonas* sp*.* |  |  | (14) |
|  |  | *Pseudomonas fluorescens* | Intestinal tract |  | (18) |
|  |  | *Pseudomonas aeruginosa* | Lungs, spleen |  | (16) |
|  | *Porphyromonas* | *Porphyromonas* sp. | Oral cavity |  | (18) |
|  | *Campylobacter* | *Campylobacter* sp. |  |  | (9) |
|  | *Prevotella* | *Prevotella intermedia* |  |  | (8) |
|  | *Fusobacterium* | *Fusobacterium simiae* |  |  | (9) |
|  | *Aggregatibacter* | *Aggregatibacter actinomycetemcomitans* |  |  | (16) |
|  | *Arthrobacter* | *Arthrobacter* sp*.* | Skin |  | (8) |
|  | *Streptococcus* | *Streptococcus* sp*.* | Intestinal tract |  | (17) |
|  |  |  | Skin |  | (12) |
| Parasite | Protozoa | *Eimeria* sp. | Intestine |  | (19) |
|  |  | *Cryptosporidium andersoni* | Gastrointestinal tract | Abdominal pain, diarrhea, vomiting |  |
|  |  | *Toxoplasma gondii* | Multiple organs | Fatal outcome |  |
|  |  | *Sarcocystis* sp*.* | Muscle tissues | N/A |  |
|  |  | *Cryptosporidium* sp*.* | Gastrointestinal tract | Abdominal pain, diarrhea, vomiting |  |
|  |  | *Babesia ailuropodae* | Red blood cells | Anemia, jaundice, hemoglobinuria | (20) |
|  |  | *Babesia* sp*.* | Red blood cells and blood |  | (21) |
|  |  |  | Red blood cells | Fever, anemia | (22) |
|  |  | *Blastocystis* sp*.* | Intestinal tract | N/A | (23) |
|  |  | *Hepatozoon* sp*.* | Blood |  |  |
|  |  | *Tyzzeria* sp*.* | Intestinal tract |  |  |
|  | Microsporidia | *Enterocytozoon bieneusi* | Small Intestine | Abdominal pain, chronic diarrhea | (19) |
|  |  |  | Intestine | Diarrhea, malnutrition | (24) |
|  | Nematoda | *Baylisascaris schroederi* | Worm: intestine, larva: various organs | Intestinal inflammation, intestinal injury, helminthic hepatitis, pneumonia, acute pancreatitis | (25) |
|  |  |  | Small intestine | Baylisascariasis | (26) |
|  |  |  |  | Emaciation, diarrhea, abdominal pain | (19) |
|  |  |  |  | Visceral larva migrans | (27) |
|  |  |  |  | Visceral larva migrans | (28) |
|  |  |  |  | N/A | (29) |
|  |  |  |  |  | (30) |
|  |  |  | Digestive tract and pancreatic ducts | Acute pancreatitis | (31) |
|  |  |  | Intestine, liver and lungs | Enteritis, inflammation, death | (32) |
|  |  |  | Small intestine, bile ducts and pancreatic ducts | Intestinal obstruction, pancreatitis, pneumonia, etc. | (33) |
|  |  |  | Small intestine | Emaciation, anorexia | (34) |
|  |  | *Baylisascaris ailuropodae* |  | Emaciation, diarrhea, abdominal pain | (19) |
|  |  |  |  | N/A |  |
|  |  | *Ancylostoma caninum* |  | Emaciation, anemia |  |
|  |  | *Toxascaris selenactis* |  | N/A |  |
|  |  | *Ancylostoma ailuropodae* |  | Emaciation, anemia |  |
|  |  |  |  | N/A | (35) |
|  |  |  | N/A |  |  |
|  |  | *Strongyloides* sp*.* |  |  | (36) |
|  |  |  | Small intestine |  | (19) |
|  |  | *Ogmoocotyle indica* |  | Intestinal mucosal hemorrhage, ulceration |  |
|  |  | *Ogmoocotyle sikae* |  |  |  |
|  |  |  | N/A | N/A | (36) |
|  |  | *Toxascaris selenarctis* |  |  |  |
|  |  |  | Small intestine |  | (19) |
|  | Ectoparasites | *Ixodes granulatus* | Body surface |  | (23) |
|  |  | *Ixodes acutitarsus* |  |  |  |
|  |  | *Ixodes ovatus* |  |  |  |
|  |  | *Haemaphysalis flava* |  |  |  |
|  |  | *Haemaphysalis apo* *Demodexses* |  |  |  |
|  |  | *Haemaphysalis hystricis* |  |  |  |
|  |  | *Haemaphysalis longicornis* |  |  |  |
|  |  | *Haemaphysalis kitaotai* |  |  |  |
|  |  | *Haemaphysalis megaspinosa* |  |  |  |
|  |  | *Haemaphysalis montgomeryi* |  |  |  |
|  |  | *Haemaphysalis warburtoni* |  |  |  |
|  |  | *Dermacentor taiwanensis* |  |  |  |
|  |  | *Haemaphysalis ailuropodae* |  |  |  |
|  |  | *Chorioptes panda* | Body surface and limbs | Severe itching, scratching, hair loss/alopecia, scab formation |  |
|  |  | *Demodex ailuropodae* | Hair follicles and sebaceous glands | Scabies-related signs |  |
|  |  | *Chaetopsylla mikado* | Body surface | Anemia/itching; possible secondary skin infection |  |
|  |  | *Chaetopsylla ailuropodae* |  |  |  |
|  | Cestoda | *Thysaniezia* sp*.* | Intestine | N/A | (19) |
|  |  | *Stilesia* sp*.* |  |  |  |

**N/A**: not available.

**References**

1. Zhao S, Hu H, Lan J, Yang Z, Peng Q, Yan L, et al. Characterization of a fatal feline panleukopenia virus derived from giant panda with broad cell tropism and zoonotic potential. Front Immunol. 2023;14.

2. Peng Q, Yang Z, Wu L, Yu P, Li Q, Lan J, et al. Evaluation of the Inactivation Efficacy of Four Disinfectants for Feline Parvovirus Derived from Giant Panda. Microorganisms. 2023;11(7).

3. Yi S, Liu S, Meng X, Huang P, Cao Z, Jin H, et al. Feline Panleukopenia Virus With G299E Substitution in the VP2 Protein First Identified From a Captive Giant Panda in China. Frontiers in Cellular and Infection Microbiology. 2022;11.

4. Gao F-S, Hu G-X, Xia X-z, Gao Y-W, Bai Y-D, Zou X-H. Isolation and identification of a canine coronavirus strain from giant pandas (Ailuropoda melanoleuca). J Vet Sci 2009;10(3).

5. Hvistendahl M. Captive pandas succumb to killer virus. Sci Total Environ. 2015:347(6223), 700–701.

6. Guo L, Yang S-l, Wang C-d, Hou R, Chen S-j, Yang X-n, et al. Phylogenetic analysis of the haemagglutinin gene of canine distemper virus strains detected from giant panda (Ailuropoda melanoleuca) and raccoon dogs (Nyctereutes procyonoides) in China. Virol J. 2013:10(1), 109.

7. Bronson E, Deem SL, Sanchez C, Murray S. Serologic response to a canarypox-vectored canine distemper virus vaccine in the giant panda (Ailuropoda melanoleuca). J Zoo Wildl Med. 2007:38(2), 363–6.

8. Yan M, Xu C, Li C, Feng Y, Duan J, Zhao K, et al. Effects of environmental disinfection on microbial population and resistance genes: A case study of the microecology within a panda enclosure. Environ Res 2023;235.

9. Ma X, Li G, Yang C, He M, Wang C, Gu Y, et al. Skin Microbiota of the Captive Giant Panda (Ailuropoda Melanoleuca) and the Distribution of Opportunistic Skin Disease-Associated Bacteria in Different Seasons. Frontiers in Veterinary Science. 2021;8.

10. Ji X, Jiang B, Feng N, Zhu L, Liang B, Liu J, et al. Complete genome sequence of a blaₙₙₘ₋₅-producing Escherichia coli DC71 assigned as ST410-O8:H9 and recovered from a captive giant panda (Ailuropoda melanoleuca) in China. J GLOB ANTIMICROB RE. 2023:32, 155–7.

11. Yan X, Yang M, Ayala JE, Li L, Zhou Y, Hou R, et al. Antimicrobial resistance, virulence genes profiles and molecular epidemiology of carbapenem-resistant Klebsiella pneumoniae strains from captive giant pandas (Ailuropoda melanoleuca). BMC Vet Res. 2024;20(1).

12. Zhou W, Zhu L, Jia M, Wang T, Liang B, Ji X, et al. Detection of Multi-Drug-ResistantEscherichia coliin a Giant Panda (Ailuropoda melanoleuca) with Extraintestinal Polyinfection. J Wildl Dis. 2018;54(3):626–30.

13. Fan S, Jiang S, Luo L, Zhou Z, Wang L, Huang X, et al. Antibiotic-Resistant Escherichia coli Strains Isolated from Captive Giant Pandas: A Reservoir of Antibiotic Resistance Genes and Virulence-Associated Genes. Vet Sci 2022;9(12).

14. Feng W, Gao C, Cui X, Yang B, He K, Huang Q, et al. Metagenome Analysis Reveals Changes in Gut Microbial Antibiotic Resistance Genes and Virulence Factors in Reintroduced Giant Pandas. Microorganisms. 2025;13(7).

15. Yang S, Gao X, Meng J, Zhang A, Zhou Y, Long M, et al. Metagenomic Analysis of Bacteria, Fungi, Bacteriophages, and Helminths in the Gut of Giant Pandas. Front Microbiol. 2018;9.

16. Wang X, Jing M, Ma Q, Lin Y, Zheng T, Yan J, et al. Oral microbiome sequencing revealed the enrichment of Fusobacterium sp., Porphyromonas sp., Campylobacter sp., and Neisseria sp. on the oral malignant fibroma surface of giant panda. Front Cell Infect Microbiol 2024;14.

17. Zhong R, Zhou Z, Liu H, Zhong Z, Peng G. Antimicrobial resistance and virulence factor gene profiles of Enterococcus spp. isolated from giant panda oral cavities. J Vet Res. 2021;65(2):147–54.

18. Xiao L-F, Li Y, Lian H, Liu X, Wen Y, Chen X, et al. Comprehensive metagenomic analysis of the giant panda’s oral microbiome reveals distinct taxonomic and functional characteristics. Animal Microbiome. 2025;7(1).

19. Li Y-h, Wang S-r, Zhou J-j, Zhou Y, Wang N, Hou Z-j. The Progress of Giant Panda Parasitic Disease (Ailuropoda melanoleuca). J Econ Anim. 2013:17(2), 117–20.

20. Xiong L, Yang G. Description and molecular characterisation of Babesia ailuropodae n. sp., a new piroplasmid species infecting giant pandas. Parasit Vectors. 2024;17(1).

21. Ma R, Yue C, Gu J, Wu W, Hou R, Huang W, et al. Efficacy of azithromycin combined with compounded atovaquone in treating babesiosis in giant pandas. Parasit Vectors. 2024;17(1).

22. Yue C, Deng Z, Qi D, Li Y, Bi W, Ma R, et al. First detection and molecular identification of Babesia sp. from the giant panda, Ailuropoda melanoleuca, in China. Parasit Vectors. 2020;13(1).

23. Li J, Karim MR, Li J, Zhang L, Zhang L. Review on parasites of wild and captive giant pandas (Ailuropoda melanoleuca): Diversity, disease and conservation impact. Int J Parasitol Parasites Wildl 2020;13:38–45.

24. Li W, Song Y, Zhong Z, Huang X, Wang C, Li C, et al. Population genetics of Enterocytozoon bieneusi in captive giant pandas of China. Parasit Vectors. 2017:10(1), 499.

25. Xu J, Gu X, Xie Y, He R, Xu J, Xiong L, et al. A novel cysteine protease inhibitor in Baylisascaris schroederi migratory larvae regulates inflammasome activation through the TLR4–ROS–NLRP3 pathway. Parasit Vectors. 2022;15(1).

26. Zhou X, Xie Y, Zhang Z-h, Wang C-d, Sun Y, Gu X-b, et al. Analysis of the genetic diversity of the nematode parasite Baylisascaris schroederi from wild giant pandas in different mountain ranges in China. Parasit Vectors. 2013:6(1), 233.

27. Lin Q, Li HM, Gao M, Wang XY, Ren WX, Cong MM, et al. Characterization of Baylisascaris schroederi from Qinling subspecies of giant panda in China by the first internal transcribed spacer (ITS-1) of nuclear ribosomal DNA. Parasitol Res 2011;110(3):1297–303.

28. Han L, Lan T, Li D, Li H, Deng L, Peng Z, et al. Chromosome‐scale assembly and whole‐genome sequencing of 266 giant panda roundworms provide insights into their evolution, adaptation and potential drug targets. Mol Ecol Resour 2021;22(2):768–85.

29. Xie Y, Zhou X, Chen L, Zhang Z, Wang C, Gu X, et al. Cloning and characterization of a novel sigma-like glutathione S-transferase from the giant panda parasitic nematode, Baylisascaris schroederi. Parasit Vectors. 2015;8(1).

30. Xie Y, Zhang Z, Wang C, Lan J, Li Y, Chen Z, et al. Complete mitochondrial genomes of Baylisascaris schroederi, Baylisascaris ailuri and Baylisascaris transfuga from giant panda, red panda and polar bear. Gene. 2011;482(1-2):59–67.

31. Qin Z, Liu S, Bai M, Geng Y, Miller DL, Zhao R, et al. First report of fatal baylisascariasis-induced acute pancreatitis in a giant panda. Parasitol Int 2021;84.

32. Xie Y, Chen S, Yan Y, Zhang Z, Li D, Yu H, et al. Potential of recombinant inorganic pyrophosphatase antigen as a new vaccine candidate against Baylisascaris schroederi in mice. Vet Res. 2013:44(1), 90.

33. Zhao G-H, Li H-M, Ryan UM, Cong M-M, Hu B, Gao M, et al. Phylogenetic study of Baylisascaris schroederi isolated from Qinling subspecies of giant panda in China based on combined nuclear 5.8S and the second internal transcribed spacer (ITS-2) ribosomal DNA sequences. Parasitol Int. 2012;61(3):497–500.

34. Hozbor DF, Sun Y, Li Y, Wu Y, Xiong L, Li C, et al. Fatty-binding protein and galectin of Baylisascaris schroederi: Prokaryotic expression and preliminary evaluation of serodiagnostic potential. Plos One. 2017;12(7).

35. Xie Y, Hoberg EP, Yang Z, Urban JF, Yang G. Ancylostoma ailuropodae n. sp. (Nematoda: Ancylostomatidae), a new hookworm parasite isolated from wild giant pandas in Southwest China. Parasit Vectors. 2017;10(1).

36. Lei Z, Xuyu Y, Hua W, Xiaodong G, Yibo H, Fuwen W. The parasites of giant pandas: individual-based measurement in wild animals. J Wildl Dis. 2011:47(1), 164–71.

**Supplementary Table 2**: Cross-species susceptible animals (non-giant pandas) to common pathogens of giant pandas

| Pathogens | Hosts | Infection ways | Infection site | Infection rate (positive number/sample number) | Mortality rate | Distribution |
| --- | --- | --- | --- | --- | --- | --- |
| *Polyomavirus* | Domestic animals: cattle(1), alpaca(2)  Pet: dog, cat(3)  Economic animals: rabbit(1)  Wild animals: parrot, goose, canary, penguin(4), primates(5), rat, field mouse, hamster(1), bat, black bear, fishes(6), birds(1) | HT | Skin, urinary system, nervous system | N/A | Goose: 100%(4) | Global distribution |
| *Canine coronavirus* | Pet: dog(7), cat(8)  Wild animals: vulpes, raccoon dog(9) | HT | Respiratory tract(main), digestive tract(part) | N/A | N/A | Global distribution |
| *Rabies virus* | Domestic animals: cattle(10), sheep and goat(11), horse, donkey(12)  Pet: dog(11), cat(12)  Wild animals: raccoon dog(10), raccoon, skunk(13), fox, (11), bat(14) | HT | Central nervous system(brain/spinal cord), salivary glandss | Stray dogs: 86.3%(12)  occipital: 57.4%(12)  Carnivora: 51.4%(12) | N/A | Global distribution |
| *Picobirnavirus-like* | Domestic animals: pig, cattle, horse(15)  Pet: dog(15)  Poultry: chicken(16)  Economic animals: rabbit(15)  Wild animals: lion, jaguar(17), snake, armadillo, pongo(15) | HT | Intestines(predominant), airway | Chicken: 3.4% (13/378)(16) | N/A | Global distribution |
| *Influenza A virus* | Domestic animals: pig, horse(18)  Poultry: duck(18), chicken, turkey, goose(19)  Wild animals: mice, ferret(18), wild water bird(19), guinea pig(20) | HT | Respiratory epithelial cells | N/A | N/A | Global distribution |
| *Rotavirus* | Domestic animals: cattle(21), sheep, goat, horse(22)  Pet: dog, cat(22)  Poultry: chicken(23), turkey(22), pigeon(24)  Economic animals: rabbit(22)  Wild animals: african lion, jaguar, mountain lion, primates(22), raccoon, civet, ungulate(24), bat(21) | HT | Small intestinal villous epithelial cells | N/A | N/A | Global distribution |
| *Feline panleukopenia virus* | Pet: cat(25)  Economic animals: breeding mink(26)  Wild animals: panthera uncia(25), raccoon, bobcat, mountain lion, skunk(27), mink(26), red fox, eurasian badger(28) | HT, VT | Digestive system  , hematopoietic system, immune system | N/A | N/A | Global distribution |
| *Canine parainfluenza virus* | Domestic animals: pig(29), cattle(30)  Pet: dog, cat(30)  Wild animals: red panda(29), hamster, guinea pig(30), rhesus monkey, crab-eating macaque(31) | HT | Airway | N/A | N/A | Global distribution |
| *Canine distemper virus* | Domestic animals: pig(32)  Pet: dog(33), cat(32)  Wild animals: raccoon, red fox(34), rhesus macaque(35), weasel, civet, rodent, sea lion, civet(32), african wild dog, lynx(36) | HT | Respiratory tract,  Digestive tract,  nervous system,  Immune system | Primates: 100%(7/7)(32)  dog: 30.0% (82/272)(32) | N/A | Global distribution |
| *Canine parvovirus* | Pet: dog(37)  Wild animals: gray wolf, suburban wolf, jackal, gray fox, Vulpes macrotis mutica, Asian raccoon dog, jungle dog, weasel family, cheetah, siberian tiger(38), crab-eating fox, crab-eating raccoon dog, white-eared opossum(39), red fox, wild dog(40) | HT, VT | Digestive tract, myocardium | N/A | Dog: 18.1%(37) | Global distribution |
| *Canine adenovirus* | Domestic animals: pig, goat, sheep(41)  Pet: dog(42)  Wild animals: rodents, primates(42), fox(43), raccoon dog, otter(44) | HT | Respiratory tract, liver | Rat: 1.9% (2/105)(44) | N/A | Global distribution |
| *Papillomavirus* | Domestic animals: cattle(45)  Pet: dog(46), cat(47)  Economic animals: rabbit(48)  Wild animals: snow leopard, mountain lion, bobcat(47), cotton rabbit, bat, hoofed animals, whales, rodents, primates(49), birds, reptile(50) | HT | Skin, mucous membrane | N/A | N/A | Global distribution |
| *Pseudorabies virus* | Domestic animals: pig(51), goat, cattle(52)  Pet: dog(51), cat(52)  Wild animals: jaguar(53), wolf, brown bear(54), fox(55), mink(52), badger(51), rodents(56), wild boar(57) | HT, VT | Central nervous system, respiratory tract | wild boar: 18% (1529/8498)(57) | Cat: 69.70%(54) | Global distribution |
| *Circovirus* | Domestic animals: pig, cattle, goat(58)  Pet: dog, cat(58)  Economic animals: rabbit(58)  Wild animals: wartpig, antelope(59), Père david's deer(60), pig, south china tiger(61), red fox, arctic fox(62), penguin(63) | HT | Multisystem | red fox: 1.4% (62)(5/115) | N/A | Global distribution |
| *Eimeria* sp. | Domestic animals: cattle(64), sheep and goat(65), alpaca, donkey, camel(66), pig(67)  Pet: dog(68)  Poultry: chicken(69)  Wild animals: roe deer(64), bat(70, 71), Vulpes lagopus, Pantholops hodgsonii(64) | HT | Digestive tract | Moschus berezovskii: 65%(64) | N/A | Global distribution |
| *Babesia* sp. | Domestic animals: cattle, goat(72), sheep(73)  Pet: dog(74)  Wild animals: Skunk, raccoon, fox, (74)otter, fox, raccoon, hare(72), shrew, rodents(73) | HT | Blood | sheep: 11.8% (11/93)(73) | N/A | Global distribution |
| *Haemaphysalis kitaokai* | Domestic animals: cattle(75), horse(76)  Wild animals: sika deer, pig, raccoon(77), rodents(78), japanese serow(79) | HT | Body surface | N/A | N/A | Asia |
| [*Enterocytozoon bieneusi*](https://spis.hnlat.com/scholar/redirect?url=https://link.springer.com/article/10.1186/s13071-016-1356-1) | Domestic animals: pig(80), cattle, sheep, horse(81)  Pet: dog, cat(82)  Poultry: chicken(81)  Economic animals: Breeding fox(83), rabbit(84)  Wild animals: pigeon(82), pig(85), macaw, myna, white-billed bulbul(86), raccoon, raccoon dog, red fox, european badger(87) | HT | Intestines, central nervous system, kidneys | Cattle: 34.4% (11/32)(81)  sheep: 25.7% (18/70)(81)  chicken: 28.9% (13/45)(81)  horse: 11.5% (3/26)(81) | N/A | Global distribution |
| *Haemaphysalis spinigera* | Domestic animals: cattle(88), sheep(89)  Pet: dog(90)  Wild animals: asian house shrew, rodents(91), leopard, jungle cat, wild dog, indian bison, mouse deer, primates(92, 93) | HT | Body surface | N/A | N/A | Asia |
| *Lungworm* | Domestic animals: sheep and goat(94), cattle(95), camel, horse(96)  Pet: cat(97), dog(96)  Wild animals: seal(98), moose, fallow deer, horse deer(95), pig, hedgepig, hare(96) | HT | Airway | Caribou: 60% (6/10)(95)  horse: 10.04% (50/498)(96)  donkey: 31.81%(21/66)(96)  mule: 24.32% (9/37)(96) | N/A | Global distribution |
| *Hepatozoon* sp. | Pet: dog(99), cat(100)  Poultry: guinea fowl(101)  Economic animals: rabbit(102)  Wild animals: grey fox, red fox, coyote(99), raccoon(100), stone marten, klipspringer, hedgepig, hare(103), rodents, marsupials(104), reptile(105) | HT | Blood cells, liver, spleen | N/A | N/A | Global distribution |
| *Toxoplasma gondii* | Domestic animals: horse(106), sheep, goat, pig, cattle, yak(107), camel(108)  Pet: dog(109), cat(110)  Poultry: chicken(107), duck, goose(109)  Economic animals: rabbit(106)  Wild animals: raccoon, skunk, bear(110), rodents, birds(106), dolphin, eurasian red squirrel(109), hare, pig(111) | HT, VT | Multisystem | cat: 70%(109)  dog: 50%(109) | N/A | Global distribution |
| *Haemaphysalis hystricis* | Domestic animals: pig, water buffalo(112)  Pet: dog(112)  Wild animals: badger(113), tiger, red muntjac, short-eared rabbit, ryukyu black rabbit(112), deer, pig(114) | HT | Body surface | N/A | N/A | Asia |
| *Haemaphysalis flava* | Domestic animals: pig, horse, sheep and goat, cattle(115)  Pet: dog(116), cat(117)  Wild animals: hedgepig(117), water deer, raccoon dog, yellow weasel, korean weasel, pig(116) | HT | Body surface | N/A | N/A | Asia |
| *Clonorchis sinensis* | Domestic animals: pig, cattle(118)  Pet: dog(119), cat(120)  Poultry: chicken, duck(118)  Economic animals: rabbit(120)  Wild animals: fishes, freshwater snails(120), weasel, fox(119) | HT | Liver, bileduct | N/A | N/A | East and Southeast Asia |
| *Haemaphysalis megaspinosa* | Domestic animals: horse(121)  Wild animals: sika deer(122), pig, raccoon(77) | HT | Body surface | N/A | N/A | Asia |
| *Strongyloides* sp. | Domestic animals: pig, horse(123), cattle, sheep and goat(124)  Pet: dog(123), cat(125)  Economic animals: rabbit(123)  Wild animals: guinea pig(123), south american raccoon(126), primates(127), arctic fox(107) | HT | Digestive tract, lungs | Polar fox: 14%(107)  Dog: 7.3% (3/41)(107) | N/A | Global distribution |
| *Ixodes granulatus* | Pet: cat(128)  Wild animals: anderson's horse mouse, black rat(129), rodents(128), tree shrew(130), musk shrew(131), japanese langur(132) | HT | Body surface | Rattus tiomanicus: 3.51% (2/57)(130)  Tupaia glis: 19.35% (6/31)(130)  Rattus rattus: 5.13% (2/39)(130)  Maxomys rajah: 25.00% (2/8)(130)  Leopoldamys sabanus: 9.09% (1/11)(130) | N/A | Asia |
| *Ixodes ovatus* | Domestic animals: horse(133)  Pet: dog(133), cat(134)  Wild animals: hare(133), forest mouse, alpine deer, gray musk shrew(135) | HT | Body surface | N/A | N/A | Asia |
| *Trichuris* sp. | Domestic animals: camel, sheep(136), pig(137)  Pet: dog(136)  Wild animals: deer(136), pig(137), uganda red gibbon(138), primates(137) | HT | Digestive tract | N/A | N/A | Global distribution |
| *Helictometra* sp. | Domestic animals: sheep, goat(139), cattle(140) | HT | Digestive tract | N/A | N/A | Global distribution |
| *Toxocara canis* | Domestic animals: pig(141)  Pet: dog(142)  Poultry: chicken(143)  Economic animals: rabbit(141)  Wild animals: rodents(144) fox, birds(141) | HT, VT | Digestive tract, liver, lungs, eyes | N/A | N/A | Global distribution |
| *Ancylostoma caninum* | Pet: dog(145), cat(146)  Wild animals: wild dog(147), rodents(148), rhesus monkey(146) | HT, VT | Digestive tract | N/A | N/A | Global distribution |
| *Demodex* sp*.* | Domestic animals: cattle, sheep and goat(149), pig, horse(150)  Pet: dog(151), cat(149)  Wild animals: house deer, european polecat, stoat(150), black-striped hamster(152), primates, bat(149), guinea pig(153) | HT, VT | Body surface | N/A | N/A | Global distribution |
| *Ixodes acutitarsus* | Wild animals: komodo dragon, tree shrew, pig(154), taiwan black bear(155) | HT | Body surface | N/A | N/A | Asia |
| *Stilesia* sp*.* | Domestic animals: sheep(156), camel(157), goat(157)  Wild animals: water antelope, black banded antelope(158) | HT | Digestive tract | N/A | N/A | Global distribution |
| *Dermacentor taiwanensis* | Domestic animals: cattle(159)  Pet: dog(160)  Wild animals: pig, black bear(155), rodents(132), red-bellied squirrel, chinese hare, ferret badger, yellow weasel(160), carnivora, bamboo chicken(161) | HT | Body surface | N/A | N/A | Taiwan |
| *Tyzzeria* sp. | Poultry: duck(162)  Economic animals: rabbit(163)  Wild animals: grey goose(164), swan goose(165), deer mouse(166), sea penguin, black goose, white-fronted goose(167) | HT | Digestive tract | N/A | N/A | Global distribution |
| *Trichinella* | Domestic animals: pig(168), horse(169), sheep(170)  Pet: dog(170), cat(170)  Poultry: chicken(171)  Economic animals: rabbit(169), bamboo rat(170)  Wild animals: birds(172), reptile, possum, insect-eating animal, armadillo, hare, odd-toed ungulate, primate(169), pig, polar bear, grizzly bear(173), marine mammal(168) | HT | Muscles, digestive tract | Sheep: 0.8% (4/500)(170)  cat: 13.3% (2/130)(170)  dogs: 16.2% (5654/34983)(170)  Cattle: 0.7% (4/500)(170) | N/A | Global distribution |
| *Blastocystis* sp. | Domestic animals: pig, cattle, sheep and goat(174)  Pet: dog, cat(174)  Poultry: chicken, duck(174)  Wild animals: ostrich, primates, snake, lizard(174), elephant, giraffe, african wild dog, whiter hino, tiger(175), rodents(176) | HT | Digestive tract | Duck: 40% (8/20)(174)  sheep: 57.9% (22/38)(174)  Horse: 12.5% (1/8)(174) |  | Global distribution |
| *Bunostomum* sp. | Domestic animals: sheep(177), cattle(178)  Wild animals: cervus elaphus sibiricusr(179) | HT | Digestive tract | Cervus elaphus sibiricus: 7.6% (10/131)(179) | N/A | Global distribution |
| *Cryptosporidium* sp. | Domestic animals: cattle, sheep and goat, pig, horse(180), donkey(181), yak, goat(182)  Pet: dog, cat(180)  Poultry: chicken(180), turkey, quail, ostrich(183)  Economic animals: rabbit(180), parrot(183)  Wild animals: deer, rodents(180), owls, mongooses, hedgepig, raccoon, bat(183), snake, lizard(184) | HT | Digestive tract, respiratory tract | N/A | N/A | Global distribution |
| *Chaetopsylla mikado* | Pet: cat(185)  Wild animals: otter, mink(185) | HT | Body surface | N/A | N/A | Asia |
| *Haemaphysalis longicornis* | Domestic animals: sheep, goat, horse, cattle(186), pig(187)  Pet: dog(188), cat(189)  Poultry: chicken(187)  Wild animals: deer(186), birds, rodents(188), grey fox, red fox(187), kangaroo, hare(190), opossum, raccoon(189) | HT | Body surface | Beaver: 55.4%(189)  white-tailed deer: 11.5%(189)  rat: 28.9%(189) | N/A | Asia, Americas |
| *Haemaphysalis aponommoides* | Domestic animals: goat(191), sheep, cattle(192), horse(193)  Pet: dog(193)  Wild animals: serow(191), pheasant with brown tail, rodents, himalayan serows, wildcats, muntjacs, flying squirrels, black bears(192) | HT | Body surface | N/A | N/A | Asia |
| *Acinetobacter nosocomialis* | Pet: dog, cat(194)  Wild animals: birds, fishes(195) | HT | Respiratory tract, urinary tract, wounds | N/A | N/A | Global distribution |
| *Proteobacteria* | Domestic animals: cattle(196), sheep(197), pig(198)  Pet: dog(198)  Poultry: chicken(196)  Wild animals: birds, reptiles, primates(196), fishes, mollusks(198) | HT | Intestines, respiratory tract, urinary tract | N/A | N/A | Global distribution |
| *Propionibacterium* | Domestic animals: cattle(199), sheep and goat(200), pig(201)  Pet: dog(200)  Poultry: chicken(202)  Economic animals: rabbit(200)  Wild animals: guinea pig(200) | HT | Body surface, respiratory tract | N/A | N/A | Global distribution |
| *Burkholderia* | Domestic animals: horse(203), goat, sheep(204)donkey, mule(205), camel(206)  Pet: dog, cat(206)  Economic animals: rabbit(206)  Wild animals: rodents(207) | HT | Respiratory tract, urinary tract | N/A | N/A | Global distribution |
| *Oxalobacter* | Domestic animals: pig, cattle, sheep and goat(208), horse(209)  Pet: cat(210)  Economic animals: rabbit(209)  Wild animals: rat(209), axolotl(211), guinea pig(208) | HT | Digestive tract | N/A | Cat: 86%(210) | Global distribution |
| *Citrobacter portucalensis* | Pet: dog, cat(212)  Poultry: chicken(213)  Wild animals:,axolotl(214), spottedturtle(215) | HT | Digestive tract, urinary tract | N/A | N/A | Global distribution |
| *Enterococcus* | Domestic animals: pig, cattle, donkey(216)  Pet: dog(217), cat(216)  Poultry: pigeon, chicken(218)  Economic animals: rabbit(218)  Wild animals: flounder, salmon, rainbow trout, goldenhead seabream(219), tufted duck, red-crested pochard, black-headed gull(220) | HT | Digestive tract, urinary tract, abdominal cavity, wound | N/A | N/A | Global distribution |
| *Salmonella enterica* | Domestic animals: pig, cattle(221)  Poultry: chicken(222), turkey(223)  Wild animals: hedgepig(224), ostrich, turkey(223), tilapia, catfish(225) | HT | Digestive tract | Lobster: 43.8% (14/32)(225)  catfish: 28.1% (9/32)(225) | N/A | Global distribution |
| *Actinobacillus* | Domestic animals: pig, sheep and goat(226), cattle, horse(227)  Pet: cat(226)  Wild animals: pig(228), ostrich(226), primates(229) | HT | Respiratory tract, body surface | N/A | N/A | Global distribution |
| *Klebsiella pneumoniae* | Domestic animals: pig(230), cattle(231)  Pet: dog, cat(232)  Poultry: chicken(230)  Economic animals: rabbit(231)  Wild animals: european sheep, snake, lizard, birds(231) | HT | Respiratory tract, urinary tract | N/A | N/A | Global distribution |
| *Hafnia alvei* | Domestic animals: horse, sheep, goat, cattle(233), camel(234)  Pet: dog(235), cat(234)  Poultry: chicken(233), duck(233), goose(234)  Wild animals: rainbow trout, cherry salmon, brown trout, sea bream(233), rhesus macaque(236), macaque, pig, père david's deer, fox, raccoon, hamster(234) | HT | Digestive tract | N/A | N/A | Global distribution |
| *Firmicutes* | Domestic animals: cattle(237), pig(238), donkey, goat, sheep, horse(239)  Pet: dog(238), cat(240)  Poultry: chicken(241)  Economic animals: rabbit(239)  Wild animals: primates, guinea pig(238), mink, fox(242), deer, raccoon dog(239), groundpig(243), kangaroo, polar bear, beaver, snake, crocodile(244), mole(245) | HT | Body surface, respiratory tract | N/A | N/A | Global distribution |
| *Flavobacterium* | Poultry: chicken(246)  Economic animals: freshwater aquaculture fish(247), salmon(248)  Wild animals: pigeon, zebra finch(246), perch, rainbow trout(248), salmon fish, catfish, gudgeon(249) | HT | Wounds, blood | N/A | N/A | Global distribution |
| *Pseudomonas* | Domestic animals: cattle, horse(250), sheep and goat(251)  Pet: dog(250)  Poultry: goose, turkey, chicken(251)  Wild animals: mouse, zebra fish(252), rat(253), mink, fox(250), otter, skunk(251) | HT | Respiratory tract, urinary tract, wounds | Cattle: 24.3%(251) | N/A | Global distribution |
| *Arthrobacter* | Domestic animals: cattle, pig(254)  Economic animals: rabbit(255) | HT | Wounds, blood | N/A | N/A | Global distribution |
| *Staphylococcus aureus* | Domestic animals: cattle(256), sheep and goat(257), horse, pig(258)  Pet: dog(256)  Poultry: chicken(256)  Economic animals: rabbit(257)  Wild animals: hedgepig, red fox, pig, moose, roe deer, european hare(259), vulture, alpine sheep, house deer, wild boar(260) | HT | Body surface, respiratory tract | Cattle: 21.2% (53/250)(256)  chicken: 8.0% (4/50)(256)  dog: 16.0% (4/25)(256)  vulture: 5.00%(260)  alpine sheep: 22.93% (36/157)(260)  mule: 19.78% (54/273)(260)  wild boar: 17.67% (126/713)(260) | N/A | Global distribution |
| *Campylobacter jejuni* | Domestic animals: cattle, sheep and goat(261), pig(262)  Pet: dog, cat(263)  Poultry: broiler, turkey(261), goose(263)  Wild animals: starling(261), bear, mule deer, ferret, hamster(263) | HT | Digestive tract | cattle: 24% (74/309)(262)  sheep: 22% (61/281)(262) | N/A | Global distribution |
| *Streptococcus* | Domestic animals: cattle, pig, horse(264), sheep(265)  Pet: dog(264), cat(266)  Poultry: chicken(264)  Economic animals: rabbit(265)  Wild animals: pigeon, koala, kangaroo, guinea pig, deer(264), freshwater dolphins, rainbow trout, tilapia(267) | HT | Respiratory tract, body surface | N/A | N/A | Global distribution |
| *Helicobacter* | Domestic animals: pig(268), horse(269), cattle, goat(269)  Pet: dog, cat(270)  Poultry: chicken(269)  Wild animals: rodents, indian fox, red panda, himalayan black bear, striped hyena, jackal, sloth bear, gray wolf, lion, tiger, common leopard(271), sea lion, gull, seal(272), rhinoceros, hippopotamus, elephant(273), pig(274) | HT | Digestive tract | Dolphin: 80% (4/5)(273)  seals: 27.3% (3/11)(273)  sea lions: 83.3%(5/6)(273) | N/A | Global distribution |
| *Moraxella* | Domestic animals: cattle(275), horse(276), goat, sheep(277)  Pet: dog, cat(278)  Wild animals: mouse, guinea pig(279), rhesus monkey, crab-eating macaque, sea lion(276), père david's deer, marsupials, marine mammals, rhesus macaque(278) | HT | Respiratory tract, conjunctiva | N/A | N/A | Global distribution |
| *Bacteroidetes* | Domestic animals: sheep and goat, pig(280), cattle(281)  Pet: dog(282)  Poultry: chicken(283), turkey(281)  Wild animals: rodents(280), rock goat(284) | HT | Abdomen, pelvis | N/A | N/A | Global distribution |
| *Proteus mirabilis* | Domestic animals: cattle(285)  Pet: dog(286), cat(287)  Poultry: chicken(288)  Economic animals: breeding dragon cat(289)  Wild animals: fox, raccoon(290) | HT | Urinary tract, wounds | Dogs: 44.4% (8/18)(286) | N/A | Global distribution |
| *Lactococcus* | Domestic animals: cattle(291), horse, pig(292), camel(293)  Pet: dog, cat(292)  Poultry: chicken(294), goose(295)  Economic animals: rainbow trout, sea bass(293), yellowtail fish, amberfish, kingfisher(292), rabbit(296)  Mullet, tilapia, grouper, yellowfin bream, japanese eel, bullfrog, roeshrimp(291), turtle(295) | HT | Digestive tract, endocardium | N/A | N/A | Global distribution |
| *Clostridium* | Domestic animals: cattle(297), sheep, goat, horse(298), horse(299)  Pet: dog, cat(298)  Poultry: broiler, turkey(300), goose(298)  Economic animals: rabbit(298)  Wild animals: partridge, raccoon, rabbit, crow(298), ostrich(299), primates, guinea pig(297) | HT | Digestive tract, wounds | N/A | N/A | Global distribution |
| *Yersinia enterocolitica* | Domestic animals: pig, cattle(301), sheep and goat, horse(302)  Pet: dog, cat(302)  Wild animals: rodents, fox, primates(302), bat, birds(301), pig, horse deer, roe deer, fallow deer(303) | HT | Digestive tract | Dog: 3.4% (1/29)(301)  pig: 12.0% (13/108)(301)  cattle: 6.0% (21/352)(301) | N/A | Global distribution |
| *Shigella* | Domestic animals: cattle(304)  Pet: dog(305)  Poultry: chicken(306)  Economic animals: rabbit(307)  Wild animals: primates(308), mouse, guinea pig(307) | HT | Digestive tract | Bovine: 1.22% (16/1311)(304)  cattle: 2.11% (10/474)(304)  beef cattle: 2.52% (8/317)(304) | N/A | Global distribution |
| *Fusarium* | Domestic animals: horse(309), pig(310)  Pet: dog(309)  Poultry: quail, chicken(311)  Economic animals: rabbit(312)  Wild animals: lizard, sea turtle, manatee(309), owl monkey(312), catfish(311), primates(310) | HT | Respiratory tract, body surface | N/A | N/A | Global distribution |
| *Aspergillus* | Domestic animals: dairy cattle(313), pig, horse(314)  Pet: dog(315)  Poultry: turkey(313)  Wild animals: birds, fishes, rodents, primates(313), penguins, harbor porpoise(315) | HT | Respiratory tract, body surface | N/A | N/A | Global distribution |
| *Cladosporium cladosporioides* | Domestic animals: sheep(316)  Pet: dog(317), cat(318)  Wild animals: panthera uncia(318), yellow-throated bunting, blue and white flycatcher, lark, barn bunting(316), sea turtle, dolphin(317) | HT | Respiratory tract, body surface |  | N/A | Global distribution |

**N/A**: not available; **HT**: Horizontal transmission; **VT**: Vertical transmission.

**References**

1. Jandrig B, Krause H, Zimmermann W, Vasiliunaite E, Gedvilaite A, Ulrich R. Hamster Polyomavirus Research: Past, Present, and Future. Viruses. 2021;13(5).

2. Dela Cruz FN, Li L, Delwart E, Pesavento PA. A novel pulmonary polyomavirus in alpacas (Vicugna pacos). Vet Microbiol. 2017;201:49–55.

3. Kamminga S, van der Meijden E, Pesavento P, Buck CB, Feltkamp MCW. Serology Identifies LIPyV as a Feline Rather than a Human Polyomavirus. Viruses. 2023;15(7).

4. Wang C-W, Chen Y-L, Mao SJT, Lin T-C, Wu C-W, Thongchan D, et al. Pathogenicity of Avian Polyomaviruses and Prospect of Vaccine Development. Viruses. 2022;14(9).

5. Ehlers B, Moens U. Genome analysis of non-human primate polyomaviruses. Infect Genet Evol. 2014;26:283–294.

6. Morris-Love J, Atwood WJ. Complexities of JC Polyomavirus Receptor-Dependent and -Independent Mechanisms of Infection. Viruses. 2022;14(6).

7. Mobasheri A. COVID-19, Companion Animals, Comparative Medicine, and One Health. Front Vet Sci. 2020;7.

8. Pratelli A, Tempesta M, Elia G, Martella V, Decaro N, Buonavoglia C. The knotty biology of canine coronavirus: A worrying model of coronaviruses' danger. Res Vet Sci. 2022;144:190–5.

9. Liu Y, Deng Y, Niu S, Zhu N, Song J, Zhang X, et al. Discovery and identification of a novel canine coronavirus causing a diarrhea outbreak in Vulpes. Sci Bull. 2023;68(21):2598–606.

10. Maki J, Guiot A-L, Aubert M, Brochier B, Cliquet F, Hanlon CA, et al. Oral vaccination of wildlife using a vaccinia–rabies-glycoprotein recombinant virus vaccine (RABORAL V-RG®): a global review. Vet Res. 2017;48(1).

11. Nahata KD, Bollen N, Gill MS, Layan M, Bourhy H, Dellicour S, et al. On the Use of Phylogeographic Inference to Infer the Dispersal History of Rabies Virus: A Review Study. Viruses. 2021;13(8).

12. Wobessi JNS, Kenmoe S, Mahamat G, Belobo JTE, Emoh CPD, Efietngab AN, et al. Incidence and seroprevalence of rabies virus in humans, dogs and other animal species in Africa, a systematic review and meta-analysis. One Health. 2021;13.

13. Markotter W, Wallace RM, Gilbert A, Slate D, Chipman R, Singh A, et al. Right Place, Wrong Species: A 20-Year Review of Rabies Virus Cross Species Transmission among Terrestrial Mammals in the United States. PLoS ONE. 2014;9(10).

14. Baker ML, Dato VM, Campagnolo ER, Long J, Rupprecht CE. A Systematic Review of Human Bat Rabies Virus Variant Cases: Evaluating Unprotected Physical Contact with Claws and Teeth in Support of Accurate Risk Assessments. PLoS ONE. 2016;11(7).

15. Rodríguez-Espinosa MJ, Rodríguez JM, Castón JR, de Pablo PJ. Mechanical disassembly of human picobirnavirus like particles indicates that cargo retention is tuned by the RNA–coat protein interaction. Nanoscale Horiz. 2023;8(12):1665–76.

16. Mondal A, Majee S. Novel bisegmented virus (picobirnavirus) of animals, birds and humans. Asian Pac J Trop Dis. 2014;4(2):154–8.

17. Gillman L, Sánchez AM, Arbiza J. Picobirnavirus in Captive Animals from Uruguay: Identification of New Hosts. Intervirology. 2013;56(1):46–9.

18. Park J, Fong Legaspi SL, Schwartzman LM, Gygli SM, Sheng Z-M, Freeman AD, et al. An inactivated multivalent influenza A virus vaccine is broadly protective in mice and ferrets. Sci Transl Med. 2022;14(653).

19. Verhagen JH, Fouchier RAM, Lewis N. Highly Pathogenic Avian Influenza Viruses at the Wild–Domestic Bird Interface in Europe: Future Directions for Research and Surveillance. Viruses. 2021;13(2).

20. Diefenbacher M, Sun J, Brooke CB. The parts are greater than the whole: the role of semi-infectious particles in influenza A virus biology. Curr Opin Virol. 2018;33:42–6.

21. Jin D-Y, Barros BdCVd, Chagas EN, Bezerra LW, Ribeiro LG, Duarte Júnior JWB, et al. Rotavirus A in wild and domestic animals from areas with environmental degradation in the Brazilian Amazon. PLoS ONE. 2018;13(12).

22. Gentsch JR, Laird AR, Bielfelt B, Griffin DD, Bányai K, et al. Serotype Diversity and Reassortment between Human and Animal Rotavirus Strains: Implications for Rotavirus Vaccine Programs. J Infect Dis. 2005;192(Suppl 1):S146–S159.

23. Alam MN, Alam MM, Nahar A, Kobayashi N, et al. Molecular Epidemiological Studies on Rotavirus Infection Causing Severe Diarrhea in Human, Animals and Poultry. Bangl J Vet Med. 2011;9(2):167–175.

24. Ghosh S, Kobayashi N. Exotic rotaviruses in animals and rotaviruses in exotic animals. VirusDis. 2014;25(2):158–72.

25. Wei J, Shi Y, Wang X, He S, Qi X, Lu R, et al. The first outbreak of feline panleukopenia virus infection in captive Pallas’s cats in Xining Wildlife Park. Front Vet Sci. 2024;11.

26. Garigliany M, Gilliaux G, Jolly S, Casanova T, Bayrou C, Gommeren K, et al. Feline panleukopenia virus in cerebral neurons of young and adult cats. BMC Vet Res. 2016;12(1).

27. Truyen U, Parrish CR. Feline panleukopenia virus: Its interesting evolution and current problems in immunoprophylaxis against a serious pathogen. Vet Microbiol. 2013;165(1-2):29–32.

28. Kapoor A, Duarte MD, Henriques AM, Barros SC, Fagulha T, Mendonça P, et al. Snapshot of Viral Infections in Wild Carnivores Reveals Ubiquity of Parvovirus and Susceptibility of Egyptian Mongoose to Feline Panleukopenia Virus. PLoS ONE. 2013;8(3).

29. Zhai J-Q, Zhai S-L, Lin T, Liu J-K, Wang H-X, Li B, et al. First complete genome sequence of parainfluenza virus 5 isolated from lesser panda. Arch Virol. 2017;162(5):1413–8.

30. Chen Z. Parainfluenza virus 5–vectored vaccines against human and animal infectious diseases. Rev Med Virol. 2018;28(2).

31. Chatziandreou N, Stock N, Young D, Andrejeva J, Hagmaier K, McGeoch DJ, et al. Relationships and host range of human, canine, simian and porcine isolates of simian virus 5 (parainfluenza virus 5). J Gen Virol. 2004;85(10):3007–16.

32. Martinez-Gutierrez M, Ruiz-Saenz J. Diversity of susceptible hosts in canine distemper virus infection: a systematic review and data synthesis. BMC Vet Res. 2016;12(1).

33. Mochizuki M, Hashimoto M, Hagiwara S, Yoshida Y, Ishiguro S, et al. Genotypes of Canine Distemper Virus Determined by Analysis of the Hemagglutinin Genes of Recent Isolates from Dogs in Japan. J Clin Microbiol. 1999;37(9):2936–2942.

34. Nikolin VM, Wibbelt G, Michler F-UF, Wolf P, East ML. Susceptibility of carnivore hosts to strains of canine distemper virus from distinct genetic lineages. Vet Microbiol. 2012;156(1-2):45–53.

35. Quintero-Gil C, Rendon-Marin S, Martinez-Gutierrez M, Ruiz-Saenz J. Origin of Canine Distemper Virus: Consolidating Evidence to Understand Potential Zoonoses. Front Microbiol. 2019;10.

36. Duque-Valencia J, Sarute N, Olarte-Castillo XA, Ruíz-Sáenz J. Evolution and Interspecies Transmission of Canine Distemper Virus—An Outlook of the Diverse Evolutionary Landscapes of a Multi-Host Virus. Viruses. 2019;11(7).

37. Alves F, Prata S, Nunes T, Gomes J, Aguiar S, Aires da Silva F, et al. Canine parvovirus: a predicting canine model for sepsis. BMC Vet Res. 2020;16(1).

38. Steinel A, Parrish CR, Bloom ME, Truyen U. Parvovirus Infections in Wild Carnivores. J Wildl Dis. 2001;37(3):594–607

39. Orozco MM, Miccio L, Enriquez GF, Iribarren FE, Gürtler RE, et al. Serologic Evidence of Canine Parvovirus in Domestic Dogs, Wild Carnivores, and Marsupials in the Argentinean Chaco. J Zoo Wildl Med. 2014;45(3):555–563.

40. Van Arkel A, Kelman M, West P, Ward MP. The relationship between reported domestic canine parvovirus cases and wild canid distribution. Heliyon. 2019;5(9).

41. Borkenhagen LK, Fieldhouse JK, Seto D, Gray GC. Are adenoviruses zoonotic? A systematic review of the evidence. Emerg Microbes Infect. 2019;8(1):1679–87.

42. Lavoie A, Liu B-h. Canine Adenovirus 2: A Natural Choice for Brain Circuit Dissection. Front Mol Neurosci. 2020;13.

43. Mira F, Puleio R, Schirò G, Condorelli L, Di Bella S, Chiaramonte G, et al. Study on the Canine Adenovirus Type 1 (CAdV-1) Infection in Domestic Dogs in Southern Italy. Pathogens. 2022;11(11).

44. Kim Y-J, Lee S-Y, Kim Y-S, Na E-J, Park J-S, Oem J-K. Genetic Characteristics of Canine Adenovirus Type 2 Detected in Wild Raccoon Dogs (Nyctereutes procyonoides) in Korea (2017–2020). Vet Sci. 2022;9(11).

45. Shah SD, Doorbar J, Goldstein RA. Analysis of Host–Parasite Incongruence in Papillomavirus Evolution Using Importance Sampling. Mol Biol Evol. 2010;27(6):1301–14.

46. Wolf J, Kist LF, Pereira SB, Quessada MA, Petek H, et al. Human papillomavirus infection: Epidemiology, biology, host interactions, cancer development, prevention, and therapeutics. Rev Med Virol. 2024;e2537.

47. Rector A, Lemey P, Tachezy R, Mostmans S, Ghim S-J, Van Doorslaer K, et al. Ancient papillomavirus-host co-speciation in Felidae. Genome Biol. 2007;8(4).

48. Cladel NM, Peng X, Christensen N, Hu J. The rabbit papillomavirus model: a valuable tool to study viral–host interactions. Philos Trans R Soc Lond B Biol Sci. 2019;374(1773).

49. Frias-De-Diego A, Jara M, Escobar LE. Papillomavirus in Wildlife. Front Ecol Evol. 2019;7.

50. Warren CJ, Van Doorslaer K, Pandey A, Espinosa JM, Pyeon D. Role of the host restriction factor APOBEC3 on papillomavirus evolution. Virus Evol. 2015;1(1).

51. Deblanc C, Oger A, Simon G, Le Potier M-F. Genetic Diversity among Pseudorabies Viruses Isolated from Dogs in France from 2006 to 2018. Pathogens. 2019;8(4).

52. Tan L, Wang K, Bai P, Zhang S, Zuo M, Shu X, et al. Host cellular factors involved in pseudorabies virus attachment and entry: a mini review. Front Vet Sci. 2023;10.

53. MÜLler T, Klupp BG, Freuling C, Hoffmann B, Mojcicz M, Capua I, et al. Characterization of pseudorabies virus of wild boar origin from Europe. Epidemiol Infect. 2010;138(11):1590–600.

54. Masot AJ, Gil M, Risco D, Jiménez OM, Núñez JI, Redondo E. Pseudorabies virus infection (Aujeszky’s disease) in an Iberian lynx (Lynx pardinus) in Spain: a case report. BMC Vet Res. 2017;13(1).

55. Zhang L, Zhong C, Wang J, Lu Z, Liu L, Yang W, et al. Pathogenesis of natural and experimental Pseudorabies virus infections in dogs. Virol J. 2015;12(1).

56. Yu X, Zhou Z, Hu D, Zhang Q, Han T, Li X, et al. Pathogenic Pseudorabies Virus, China, 2012. Emerg Infect Dis. 2014;20(1):102–4.

57. Pedersen K, Bevins SN, Baroch JA, Cumbee JC, Chandler SC, Woodruff BS, et al. Pseudorabies in Feral Swine in the United States, 2009–2012. J Wildl Dis. 2013;49(3):709–13.

58. Wen L, Zhu J, Zhang F, Xiao Q, Xie J, He K. Interaction of porcine circovirus-like virus P1 capsid protein with host proteins. BMC Vet Res. 2021;17(1).

59. Molini U, Coetzee LM, Hemberger MY, Khaiseb S, Cattoli G, Dundon WG, et al. The Oryx Antelope (Oryx gazella): An Unexpected Host for Porcine Circovirus-2 (PCV-2). Pathogens. 2021;10(11).

60. Fisher M, Harrison TMR, Nebroski M, Kruczkiewicz P, Rothenburger JL, Ambagala A, et al. Discovery and comparative genomic analysis of elk circovirus (ElkCV), a novel circovirus species and the first reported from a cervid host. Sci Rep. 2020;10(1).

61. Cui X, Fan K, Liang X, Gong W, Chen W, He B, et al. Virus diversity, wildlife-domestic animal circulation and potential zoonotic viruses of small mammals, pangolins and zoo animals. Nat Commun. 2023;14(1).

62. Franzo G, Menandro ML, Tucciarone CM, Barbierato G, Crovato L, Mondin A, et al. Canine Circovirus in Foxes from Northern Italy: Where Did It All Begin? Pathogens. 2021;10(8).

63. Roberts LC, Molini U, Coetzee LM, Khaiseb S, Roux J-P, Kemper J, et al. Is Penguin Circovirus Circulating Only in the Antarctic Circle? Lack of Viral Detection in Namibia. Animals. 2023;13(9).

64. Bangoura B, Bhuiya MAI, Kilpatrick M. Eimeria infections in domestic and wild ruminants with reference to control options in domestic ruminants. Parasitol Res. 2022;121(8):2207–32.

65. Diao N-C, Zhao B, Chen Y, Wang Q, Chen Z-Y, Yang Y, et al. Prevalence of Eimeria Spp. Among Goats in China: A Systematic Review and Meta-Analysis. Front Cell Infect Microbiol. 2022;12.

66. Dubey JP. A review of coccidiosis in South American camelids. Parasitol Res. 2018;117(7):1999–2013.

67. Gong Q-L, Zhao W-X, Wang Y-C, Zong Y, Wang Q, Yang Y, et al. Prevalence of coccidia in domestic pigs in China between 1980 and 2019: a systematic review and meta-analysis. Parasites Vectors. 2021;14(1).

68. Dubey JP, Lindsay DS. Coccidiosis in dogs—100 years of progress. Vet Parasitol. 2019;266:34–55.

69. Matsubayashi M, Shibahara T, Matsuo T, Hatabu T, Yamagishi J, Sasai K, et al. Morphological and molecular identification of Eimeria spp. in breeding chicken farms of Japan. J Vet Med Sci. 2020;82(5):516–9.

70. Murakoshi F, Recuenco FC, Omatsu T, Sano K, Taniguchi S, Masangkay JS, et al. Detection and molecular characterization of Cryptosporidium and Eimeria species in Philippine bats. Parasitol Res. 2016;115(5):1863–9.

71. Tan Z, Gonzalez G, Sheng J, Wu J, Zhang F, Xu L, et al. Extensive Genetic Diversity of Polyomaviruses in Sympatric Bat Communities: Host Switching versus Coevolution. J Virol. 2020;94(9):e02101-19.

72. Yabsley MJ, Shock BC. Natural history of Zoonotic Babesia: Role of wildlife reservoirs. Int J Parasitol Parasites Wildl. 2013;2:18–31.

73. Gray A, Capewell P, Loney C, Katzer F, Shiels BR, Weir W. Sheep as Host Species for Zoonotic Babesia venatorum, United Kingdom. Emerg Infect Dis. 2019;25(12):2257–60.

74. Goethert HK, Telford SR. What is Babesia microti? Parasitology. 2003;127(4):301–9.

75. Yamane I, Nishiguchi A, Kobayashi S, Zeniya Y. Cross-Sectional Survey of Ixodid Tick Species on Grazing Cattle in Japan. Exp Appl Acarol. 2006;38(1):67–74.

76. Zhang YK, Zhang XY, Liu JZ. Ticks (Acari: Ixodoidea) in China: Geographical distribution, host diversity, and specificity. Arch Insect Biochem Physiol. 2019;102(3).

77. Stevenson B, Furuno K, Lee K, Itoh Y, Suzuki K, Yonemitsu K, et al. Epidemiological study of relapsing fever borreliae detected in Haemaphysalis ticks and wild animals in the western part of Japan. PLoS ONE. 2017;12(3).

78. Matsuda Y, Okajima M, Fujii Y, Izumi F, Takahashi MR, Iwatake Y, et al. Diversity of piroplasma species in small rodents and ticks captured in suburbs of Gifu City, central Japan. J Vet Med Sci. 2025;87(1):43–51.

79. Hoogstraal H. Haemaphysalis (Alloceraea) kitaokai sp. n. of Japan, and Keys to Species in the Structurally Primitive Subgenus Alloceraea Schulze of Eurasia (Ixodoidea, Ixodidae). J Parasitol. 1969;55(1):211–221.

80. Reetz J, Nöckler K, Reckinger S, Vargas MM, Weiske W, Broglia A. Identification of Encephalitozoon cuniculi genotype III and two novel genotypes of Enterocytozoon bieneusi in swine. Parasitol Int. 2009;58(3):285–92.

81. Mohammad Rahimi H, Mirjalali H, Zali MR. Molecular epidemiology and genotype/subtype distribution of Blastocystis sp., Enterocytozoon bieneusi, and Encephalitozoon spp. in livestock: concern for emerging zoonotic infections. Sci Rep. 2021;11(1).

82. Santín M, Fayer R. Microsporidiosis: Enterocytozoon bieneusi in domesticated and wild animals. Res Vet Sci. 2011;90(3):363–71.

83. Zhang X-X, Cong W, Lou Z-L, Ma J-G, Zheng W-B, Yao Q-X, et al. Prevalence, risk factors and multilocus genotyping of Enterocytozoon bieneusi in farmed foxes (Vulpes lagopus), Northern China. Parasites Vectors. 2016;9(1).

84. Deng L, Chai Y, Xiang L, Wang W, Zhou Z, Liu H, et al. First identification and genotyping of Enterocytozoon bieneusi and Encephalitozoon spp. in pet rabbits in China. BMC Vet Res. 2020;16(1).

85. Němejc K, Sak B, Květoňová D, Hanzal V, Janiszewski P, Forejtek P, et al. Prevalence and diversity of Encephalitozoon spp. and Enterocytozoon bieneusi in wild boars (Sus scrofa) in Central Europe. Parasitol Res. 2013;113(2):761–7.

86. Tavalla M, Mardani-Kateki M, Abdizadeh R, Soltani S, Saki J. Molecular diagnosis of potentially human pathogenic Enterocytozoon bieneusi and Encephalitozoon species in exotic birds in Southwestern Iran. J Infect Public Health. 2018;11(2):192–6.

87. Perec-Matysiak A, Leśniańska K, Buńkowska-Gawlik K, Merta D, Popiołek M, Hildebrand J. Zoonotic Genotypes of Enterocytozoon bieneusi in Wild Living Invasive and Native Carnivores in Poland. Pathogens. 2021;10(11).

88. Balasubramanian R, Yadav PD, Sahina S, Arathy Nadh V. Distribution and prevalence of ticks on livestock population in endemic area of Kyasanur forest disease in Western Ghats of Kerala, South India. J Parasit Dis. 2019;43(2):256–62.

89. Kumar K, Balakrishnan N, Sharma AK. Studies on the Vertical Distribution of Ticks of Domestic Animals and Their Public Health Importance in Nilgiri Hills and Adjoining Areas of Tamil Nadu State (India). International Journal of Zoology. 2014;2014:1–6.

90. Bandaranayaka KO, Dissanayake UI, Rajakaruna RS, et al. Diversity and geographic distribution of dog tick species in Sri Lanka and the life cycle of brown dog tick, Rhipicephalus sanguineus under laboratory conditions. Res Square. 2022

91. Yathramullage S, Dissanayake U, Rajakaruna RS, Meegaskumbura S. New records of small mammal hosts for five ectoparasite species from Sri Lanka. Ceylon J Sci. 2016;45(3).

92. Muraleedharan K. Wildlife Arthropods of Karnataka with Special Reference to KFD Endemic Area of Shivamogga District: Those Parasitic on Small and Large Mammals. Vet Res Int. 2016;4(4):114–123.

93. Kumar KGA, Ravindran R, Johns J, Chandy G, Rajagopal K, et al. Ixodid Tick Vectors of Wild Mammals and Reptiles of Southern India. J Arthropod-Borne Dis. 2018;12(3):276–285.

94. Bentounsi B, Cabaret J. Small-Lungworm (Protostrongylidae) Infections in Relation to Meat Sheep Breeds, Mediterranean Climates, and Anthelmintic Regimens. Vet Sci. 2025;12(5).

95. Kowal J, Kornaś S, Nosal P, Basiaga M, Wajdzik M, et al. Lungworm (Nematoda: Protostrongylidae) infection in wild and domestic ruminants from Małopolska region of Poland. Ann Parasitol. 2016;62(1):63–66.

96. Mohtasebi S, Sazmand A, Zafari S, Verocai GG, Otranto D. Lungworms of Non-Ruminant Terrestrial Mammals and Humans in Iran. Pathogens. 2023;12(6).

97. Di Cesare A, Gueldner EK, Traversa D, Veronesi F, Morelli S, Crisi PE, et al. Seroprevalence of antibodies against the cat lungworm Aelurostrongylus abstrusus in cats from endemic areas of Italy. Vet Parasitol. 2019;272:13–6.

98. van Wijngaarden MFA, Geut MIM, Vernooij JCM, Ijsseldijk LL, Tobias TJ. Determinants of mortality of juvenile harbour seals (Phoca vitulina) infected with lungworm submitted to a Dutch seal rehabilitation centre. Int J Parasitol Parasites Wildl. 2021;14:1–6.

99. Baker E, Jensen A, Miller D, Garrett KB, Cleveland CA, Brown J, et al. Hepatozoon spp. infection in wild canids in the eastern United States. Parasites Vectors. 2023;16(1).

100. Thomas R, Santodomingo A, Saboya-Acosta L, Quintero-Galvis JF, Moreno L, Uribe JE, et al. Hepatozoon (Eucoccidiorida: Hepatozoidae) in wild mammals of the Americas: a systematic review. Parasites Vectors. 2024;17(1).

101. Tembe D, Malatji MP, Mukaratirwa S. Occurrence, Prevalence, and Distribution of Haemoparasites of Poultry in Sub-Saharan Africa: A Scoping Review. Pathogens. 2023;12(7).

102. Allen KE, Johnson EM, Little SE. Hepatozoon spp Infections in the United States. Vet Clin North Am Small Anim Pract. 2011;41(6):1221–38.

103. Uiterwijk M, Vojta L, Šprem N, Beck A, Jurković D, Kik M, et al. Diversity of Hepatozoon species in wild mammals and ticks in Europe. Parasites Vectors. 2023;16(1).

104. Weck BC, Serpa MCA, Ramos VN, Luz HR, Costa FB, Ramirez DG, et al. Novel genotypes of Hepatozoon spp. in small mammals, Brazil. Parasites Vectors. 2022;15(1).

105. Perles L, Roque ALR, D’Andrea PS, Lemos ERS, Santos AF, Morales AC, et al. Genetic diversity of Hepatozoon spp. in rodents from Brazil. Sci Rep. 2019;9(1).

106. Tenter AM, Heckeroth AR, Weiss LM, et al. Toxoplasma gondii: from animals to humans. Int J Parasitol. 2000;30(12-13):1217–1258.

107. Dillard KJ, Saari SAM, Anttila M. Strongyloides stercoralis infection in a Finnish kennel. Acta Vet Scand. 2007;49(1).

108. Gebremedhin EZ, Tadesse G. A meta-analysis of the prevalence of Toxoplasma gondii in animals and humans in Ethiopia. Parasites Vectors. 2015;8(1).

109. Webster JP. The Effect of Toxoplasma gondii on Animal Behavior: Playing Cat and Mouse. Schizophr Bull. 2007;33(3):752–6.

110. Sibley LD, Khan A, Ajioka JW, Rosenthal BM. Genetic diversity of Toxoplasma gondii in animals and humans. Philos Trans R Soc Lond B Biol Sci. 2009;364(1530):2749–61.

111. Tenter AM. Toxoplasma gondii in animals used for human consumption. Mem Inst Oswaldo Cruz. 2009:104(2), 364–9.

112. Thekisoe OMM, Honda T, Fujita H, Battsetseg B, Hatta T, et al. A trypanosome species isolated from naturally infected Haemaphysalis hystricis ticks in Kagoshima Prefecture, Japan. Vet Parasitol. 2007;148(3-4):273–282.

113. Tian J, Ge M, Xu H, Wu T, Yu B, Lei C. The complete mitochondrial genome and phylogenetic analysis of Haemaphysalis hystricis (Parasitiformes: Ixodidae). Mitochondrial DNA Part B. 2019;4(1):1049–50.

114. Jongejan F, Su B-L, Yang H-J, Berger L, Bevers J, Liu P-C, et al. Molecular evidence for the transovarial passage of Babesia gibsoni in Haemaphysalis hystricis (Acari: Ixodidae) ticks from Taiwan: a novel vector for canine babesiosis. Parasites Vectors. 2018;11(1).

115. Li D, Liu L, Liu Z-l, Tian Y, Gao X, Cheng T-y. What are the main proteins in the hemolymph of Haemaphysalis flava ticks? Front Vet Sci. 2024;11.

116. Kakuda H, Shiraishi S, Uchida T. Seasonal Fluctuations of Populations and Effects of Temperatures on Development and Growth in the Tick, Haemaphysalis flava. J Fac Agric, Kyushu Univ. 1990;35(1/2):17–26.

117. Sang Min K, Park Jie e, Song Dae K, Jeong Jun Y, Hong CE, Shin H, et al. Microbiome Composition of Haemaphysalis flava in Korea and Diversity Analysis Based on Region, Developmental Stage, and Sex. Entomol Res. 2025;55(3).

118. Ittiprasert W, Pakharukova MY, Lishai EA, Zaparina O, Baginskaya NV, Hong S-J, et al. Opisthorchis viverrini, Clonorchis sinensis and Opisthorchis felineus liver flukes affect mammalian host microbiome in a species-specific manner. PLoS Negl Trop Dis. 2023;17(2).

119. Na B-K, Pak JH, Hong S-J. Clonorchis sinensis and clonorchiasis. Acta Trop. 2020;203.

120. Tang Z-L, Huang Y, Yu X-B. Current status and perspectives of Clonorchis sinensis and clonorchiasis: epidemiology, pathogenesis, omics, prevention and control. Infect Dis Poverty. 2016;5(1).

121. YbaÑEz AP, Sato F, Nambo Y, Fukui T, Masuzawa T, Ohashi N, et al. Survey on Tick-Borne Pathogens in Thoroughbred Horses in the Hidaka District, Hokkaido, Japan. J Vet Med Sci. 2013;75(1):11–5.

122. Shimizu K, Shimozuru M, Yamanaka M, Ito G, Nakao R, Tsubota T. Seasonal infestation patterns of ticks on Hokkaido sika deer (Cervus nippon yesoensis). Parasitology. 2024;151(12):1317–25.

123. Brygadyrenko VV, Boyko OO, Gugosyan YA. Morphological variation of four species of Strongyloides (Nematoda, Rhabditida) parasitising various mammal species. Biosyst Divers. 2019;27(1):85–98.

124. Viney M. Strongyloides. Parasitology. 2016;144(3):259–62.

125. Wulcan JM, Dennis MM, Ketzis JK, Bevelock TJ, Verocai GG. Strongyloides spp. in cats: a review of the literature and the first report of zoonotic Strongyloides stercoralis in colonic epithelial nodular hyperplasia in cats. Parasites Vectors. 2019;12(1).

126. Darling ST. Strongyloides Infections in Man and Animals in the Isthmian Canal Zone. J Exp Med. 1911;14(1):1–24.

127. Nosková E, Sambucci KM, Petrželková KJ, Červená B, Modrý D, Pafčo B. Strongyloides in non-human primates: significance for public health control. Philos Trans R Soc Lond B Biol Sci. 2023;379(1894).

128. Doi K, Tokuyoshi M, Morishima K, Kogi K, Watari Y. Differential Tick-Infestation Rate between Rattus norvegicus and R. rattus, with the First Records of the Ixodid Tick Ixodes granulatus and Its Infestation in Rodents, Free-Ranging Cats, and Humans from Mikura-Shima Island, Japan. Mamm Study. 2022;47(4).

129. Paperna I. The tick Ixodes granulatus infests Rattus rattus populating a small island offshore of Singapore. Parasite. 2006;13(1):83–4.

130. Ishak SN, Yusof MA, Md-Nor S, Mohd Sah SA, Lim FS, Khoo JJ, et al. Prevalence of on-host ticks (Acari: Ixodidae) in small mammals collected from forest near to human vicinity in Selangor, Malaysia. Syst Appl Acarol. 2018;23(8).

131. Jansen A, La Scola B, Raoult D, Lierz M, Wichmann O, Stark K, et al. Antibodies against Rickettsia spp. in Hunters, Germany. Emerg Infect Dis. 2008;14(12):1961–3.

132. Hornok S, Kontschán J, Keve G, Takács N, Van Nguyen D, Ho KNP, et al. First report of Haemaphysalis bispinosa, molecular-geographic relationships of Ixodes granulatus and a new Dermacentor species from Vietnam. Parasites Vectors. 2025;18(1).

133. Morel PC. Identité d’Ixodes ovatus Neumann, 1899 (Acariens, Ixodoidea). Ann Parasitol Hum Comp. 2017;38(6):925–8.

134. Brissette CA, Chao L-L, Liu L-L, Ho T-Y, Shih C-M. First Detection and Molecular Identification of Borrelia garinii Spirochete from Ixodes ovatus Tick Ectoparasitized on Stray Cat in Taiwan. PLoS ONE. 2014;9(10).

135. Yano Y, Takada N, Ishiguro F. Location and Ultrastructure of Borrelia japonica in Naturally Infected Ixodes ovatus and Small Mammals. Microbiol Immunol. 2013;41(1):13–9.

136. Hansen TVA, Nejsum P, Olsen A, Thamsborg SM. Genetic variation in codons 167, 198 and 200 of the beta-tubulin gene in whipworms (Trichuris spp.) from a range of domestic animals and wildlife. Vet Parasitol. 2013;193(1-3):141–9.

137. Cutillas C, Callejón R, de Rojas M, Tewes B, Ubeda JM, Ariza C, et al. Trichuris suis and Trichuris trichiura are different nematode species. Acta Trop. 2009;111(3):299–307.

138. Righini N, Barelli C, Gonzalez-Astudillo V, Mundry R, Rovero F, Hauffe HC, et al. Altitude and human disturbance are associated with helminth diversity in an endangered primate, Procolobus gordonorum. PLoS ONE. 2019;14(12).

139. Alfatlawi MA, Ismail YK, Ali MJ, Karawan AC, Ibadi IN. Molecular differentiation of Thysaniezia (Helictometra) giardi and Moniezia species based on 18s rRNA gene in small ruminants. Iraqi J Vet Sci. 2021;35(1):105–8.

140. Southwell T. Notes on the Anatomy of Stilesia Hepatica, and on the Genera of the Sub-Family Thysanosominae (Including Avitellininae). Ann Trop Med Parasitol. 2016;23(1):47–66.

141. Schnieder T, Laabs E-M, Welz C. Larval development of Toxocara canis in dogs. Vet Parasitol. 2011;175(3-4):193–206.

142. Schwartz R, Bidaisee S, Fields PJ, Macpherson MLA, Macpherson CNL. The epidemiology and control of Toxocara canis in puppies. Parasite Epidemiol Control. 2022;16.

143. Taira K, Saeed I, Permin A, Kapel CMO. Zoonotic risk of Toxocara canis infection through consumption of pig or poultry viscera. Vet Parasitol. 2004;121(1-2):115–24.

144. Zhu X-Q, Korhonen PK, Cai H, Young ND, Nejsum P, von Samson-Himmelstjerna G, et al. Genetic blueprint of the zoonotic pathogen Toxocara canis. Nat Commun. 2015;6(1).

145. Mawrie UG, Kharkongor R, Valladares MM, Kepha S, Ajjampur SSR, et al. The occurrence of cross-host species soil-transmitted helminth infections in humans and domestic/livestock animals: A systematic review. PLOS Glob Public Health. 2025;5(8):e0004614.

146. Lee KT, Little MD, Beaver PC. Intracellular (Muscle-Fiber) Habitat of Ancylostoma caninum in Some Mammalian Hosts. J Parasitol. 1975;61(4):589–598.

147. Smout FA, Thompson RCA, Skerratt LF. First report of Ancylostoma ceylanicum in wild canids. Int J Parasitol Parasites Wildl. 2013;2:173–7.

148. Silva R, Leite Diniz F, Aguiar de Oliveira P, Alves de Farias L. Particularidades do Ancylostoma caninum: Revisão. Pubvet. 2020;15(01).

149. Izdebska JN, Rolbiecki L. The Biodiversity of Demodecid Mites (Acariformes: Prostigmata), Specific Parasites of Mammals with a Global Checklist and a New Finding for Demodex sciurinus. Diversity. 2020;12(7).

150. Nutting WB, Kettle PR, Tenquist JD, Whitten LK. Hair follicle mites (Demodex spp.) in New Zealand. N Z J Zool. 2010;2(2):219–22.

151. Sivajothi S, Sudhakara Reddy B, Rayulu VC. Demodicosis caused by Demodex canis and Demodex cornei in dogs. J Parasit Dis. 2013;39(4):673–6.

152. Izdebska JN, Cydzik K. Occurrence of Demodex spp. (Acari, Demodecidae) in the striped field mouse Apodemus agrarius (Rodentia, Muridae) in Poland. Wiadomosci Parazytologiczne. 2010;56(1):59–61.

153. Schönfelder J, Henneveld K, Schönfelder A, Hein J, Müller R, et al. Concurrent infestation of Demodex caviae and Chirodiscoides caviae in a guinea pig. Tierärztl Prax. 2010;38(K):28–30.

154. Warburton C. On Five New Species of Ticks (Arachnida Ixodoidea). Parasitology. 1933;24(4):558–569.

155. Tsai Y-L, Wechtaisong W, Lee T-R, Chang C-H, Yu P-H, Hwang M-H. Hematological and plasma profiles and ticks and tick-borne pathogens in wild Formosan black bears (Ursus thibetanus formosanus). Parasites Vectors. 2024;17(1).

156. Louw JP. Diagnosis, distribution and prevalence of Stilesia globipunctata (Rivolta 1874) in sheep in the Overberg Region of the southern Western Cape Province. J S Afr Vet Assoc. 1995;66(4):244–246.

157. Abed HH, Fadhil AI, Alhaboubi AR, Farj AA. Genetic confirmation for morphological identification of Stilesia globipunctata in camel in Iraq. Iraqi J Vet Sci. 2023;37(3):719–724.

158. Van Wyk IC, Boomker J. Parasites of South African wildlife. XIX. The prevalence of helminths in some common antelopes, warthogs and a bushpig in the Limpopo province, South Africa. Onderstepoort J Vet Res. 2011;78(1).

159. Zhang X, Li H-Y, Shao J-W, Pei M-C, Cao C, Huang F-Q, et al. Genomic characterization and phylogenetic analysis of a novel Nairobi sheep disease genogroup Orthonairovirus from ticks, Southeastern China. Front Microbiol. 2022;13.

160. Apanaskevich MA, Apanaskevich DA. Reinstatement of Dermacentor bellulus (Acari: Ixodidae) as a Valid Species Previously Confused with D. taiwanensis and Comparison of All Parasitic Stages. J Med Entomol. 2015;52(4):573–95.

161. Hoogstraal H, Wassef HY, Santana FJ, Kuntz RE. Dermacentor (Indocentor) taiwanensis (Acari: Ixodoidea: Ixodidae): Hosts and Distribution in Taiwan and Southern Japan. J Med Entomol. 1986;23(3):286–288.

162. Pecka Z. The life cycle of Eimeria danailovi from ducks. Folia Parasitol. 1992;39(1):13–18.

163. Arias-Pacheco C, Perin PP, Oliveira WJ, de Souza Pollo A, Benatti D, Mendonça TO, et al. Helminth parasites of the invasive European brown hare (Lepus europaeus) in Brazil: the first report of Bunostomum trigonocephalum in a hare. Parasitol Res. 2024;123(12).

164. Berto BP, Teixeira M, Lopes CWG. Tyzzeria parvula (Kotlan, 1933) Klimes, 1963 (Apicomplexa: Eimeriidae) in the greylag goose (Anser anser Linnaeus, 1758) in southeastern Brazil. Rev Bras Parasitol Vet. 2007;16(3):156–158.

165. Oliveira MS, Ortúzar-Ferreira CN, Lima VM, Cardozo SV, Lopes CWG, Berto BP. A systematic review of the genus Tyzzeria Allen, 1936 (Chromista: Apicomplexa), including the molecular phylogenetic position of Tyzzeria parvula (Kotlán, 1933) Klimeš, 1963. Parasitol Int. 2025;106.

166. Levine ND, Ivens V. Eimeria and Tyzzeria (Protozoa: Eimeriidae) from deermice (Peromyscus spp.) in Illinois. J Parasitol. 1960;46(2):207–212.

167. Brown MA, Ball SJ, Snow KR. Coccidian parasites of British wild birds. J Nat Hist. 2010;44(43-44):2669–2691.

168. Pozio E. Factors affecting the flow among domestic, synanthropic and sylvatic cycles of Trichinella. Vet Parasitol. 2000;93(3-4):241-262.

169. Pozio E. The broad spectrum of Trichinella hosts: From cold- to warm-blooded animals. Vet Parasitol. 2005;132(1-2):3–11.

170. Wang ZQ, Cui J, Shen LJ. The epidemiology of animal trichinellosis in China. Vet J. 2007;173(2):391–8.

171. Pozio E, La Rosa G, Rossi P, Murrell KD. Biological characterization of Trichinella isolates from various host species and geographical regions. J Parasitol. 1992;78(4):647–653.

172. Pozio E. World distribution of Trichinella spp. infections in animals and humans. Vet Parasitol. 2007;149(1-2):3–21.

173. Pozio E. Trichinella spp. imported with live animals and meat. Vet Parasitol. 2015;213(1-2):46–55.

174. Rauff-Adedotun AA, Mohd Zain SN, Farah Haziqah MT. Current status of Blastocystis sp. in animals from Southeast Asia: a review. Parasitol Res. 2020;119(11):3559–70.

175. Parkar U, Traub RJ, Vitali S, Elliot A, Levecke B, Robertson I, et al. Molecular characterization of Blastocystis isolates from zoo animals and their animal-keepers. Vet Parasitol. 2010;169(1-2):8–17.

176. Deng L, Chai Y, Zhou Z, Liu H, Zhong Z, Hu Y, et al. Epidemiology of Blastocystis sp. infection in China: a systematic review. Parasite. 2019;26.

177. Belle EA. The effect of microenvironment on the free-living stages of Bunostomum trigonocephalum. Can J Zool. 1959;37(2):289–298.

178. Jex AR, Waeschenbach A, Hu M, van Wyk JA, Beveridge I, Littlewood DTJ, et al. The mitochondrial genomes of Ancylostoma caninum and Bunostomum phlebotomum – two hookworms of animal health and zoonotic importance. BMC Genomics. 2009;10(1).

179. Abdybekova AM, Sultanov AA, Dzhusupbekova NM, Abdibayeva AA, Zhaksylykova AA, Kerimbaeva RA, et al. Parasites of farmed marals in Kazakhstan. Small Rumin Res. 2017;153:142–5.

180. Angus KW. Cryptosporidiosis in man, domestic animals and birds: a review. J R Soc Med. 1983;76(1):62–70.

181. Li F, Su J, Chahan B, Guo Q, Wang T, Yu Z, et al. Different distribution of Cryptosporidium species between horses and donkeys. Infect Genet Evol. 2019;75.

182. Gao J-F, Zhao Q, Liu G-H, Zhang Y, Zhang Y, Wang W-T, et al. Comparative analyses of the complete mitochondrial genomes of the two ruminant hookworms Bunostomum trigonocephalum and Bunostomum phlebotomum. Gene. 2014;541(2):92–100.

183. El-Alfy E-S, Nishikawa Y. Cryptosporidium species and cryptosporidiosis in Japan: a literature review and insights into the role played by animals in its transmission. J Vet Med Sci. 2020;82(8):1051–67.

184. Danišová O, Halánová M, Valenčáková A, Luptáková L. Sensitivity, specificity and comparison of three commercially available immunological tests in the diagnosis of Cryptosporidium species in animals. Braz J Microbiol. 2018;49(1):177–83.

185. Medvedev SG, Seryodkin IV. Fleas (Siphonaptera) of Carnivores (Mammalia, Carnivora) of the Russian Far East. Entomol Rev. 2019;99(1):70–7.

186. Jiang J, An H, Lee JS, O'Guinn ML, Kim HC, et al. Molecular characterization of Haemaphysalis longicornis-borne rickettsiae, Republic of Korea and China. Infect Genet Evol. 2018

187. Egizi A, Bulaga‐Seraphin L, Alt E, Bajwa WI, Bernick J, Bickerton M, et al. First glimpse into the origin and spread of the Asian longhorned tick, Haemaphysalis longicornis, in the United States. Zoonoses Public Health. 2020;67(6):637–50.

188. Raghavan RK, Barker SC, Cobos ME, Barker D, Teo EJM, Foley DH, et al. Potential Spatial Distribution of the Newly Introduced Long-horned Tick, Haemaphysalis longicornis in North America. Sci Rep. 2019;9(1).

189. Tufts DM, Goodman LB, Benedict MC, Davis AD, VanAcker MC, Diuk-Wasser M. Association of the invasive Haemaphysalis longicornis tick with vertebrate hosts, other native tick vectors, and tick-borne pathogens in New York City, USA. Int J Parasitol. 2021;51(2-3):149–57.

190. Hoogstraal H, Roberts FHS, Kohls GM, Tipton VJ. Review of Haemaphysalis (Kaiseriana) longicornis Neumann (resurrected) of Australia, New Zealand, New Caledonia, Fiji, Japan, Korea, and northeastern China and USSR, and its parthenogenetic and bisexual populations (Ixodoidea, Ixodidae). J Parasitol. 1968;54(6):1197–1213.

191. Hoogstraal H. Haemaphysalis nepalensis sp. n. from a Himalayan rodent and man, and description of the male of H. aponommoides Warburton (n. comb.) (Ixodoidea, Ixodidae). J Parasitol. 1962;48(2):195–203.

192. Pun SK, Guglielmone AA, Tarragona EL, Nava S, Maharjan M. Ticks (Acari: Ixodidae) of Nepal: First record of Amblyomma varanense (Supino), with an update of species list. Ticks Tick-borne Dis. 2018;9(3):526–34.

193. Hoogstraal H, Mitchell RM. Haemaphysalis (Alloceraea) aponommoides Warburton (Ixodoidea Ixodidae), description of immature stages, hosts, distribution, and ecology in India, Nepal, Sikkim, and China. J Parasitol. 1971: 635-645.

194. Wong D, Nielsen TB, Bonomo RA, Pantapalangkoor P, Luna B, Spellberg B. Clinical and Pathophysiological Overview of Acinetobacter Infections: a Century of Challenges. Clin Microbiol Rev. 2017;30(1):409–47.

195. Almasaudi SB. Acinetobacter spp. as nosocomial pathogens: Epidemiology and resistance features. Saudi J Biol Sci. 2018;25(3):586–96.

196. Ma Z, Shi P. Critical complex network structures in animal gastrointestinal tract microbiomes. Anim Microbiome. 2024;6(1).

197. Gallardo Paffetti M, Azócar-Aedo L, Parra-Vizcaíno A, Larraín JM, Díaz A. Impact of maternal diet and pregnancy type on the abundance of zoonotic bacteria (Firmicutes and Proteobacteria) in sheep feces and wool. Front Anim Sci. 2025;6.

198. Kersters K, De Vos P, Gillis M, Swings J. Proteobacteria. eLS. 2006

199. Thierry A, Deutsch S-M, Falentin H, Dalmasso M, Cousin FJ, Jan G. New insights into physiology and metabolism of Propionibacterium freudenreichii. Int J Food Microbiol. 2011;149(1):19–27.

200. Webster GF, Ruggieri MR, KJ M. Correlation of Propionibacterium acnes Populations with the Presence of Triglycerides on Nonhuman Skin. Appl Environ Microbiol. 1981;41(5):1269-1270.

201. Mantere-Alhonen S. Propionibacteria used as probiotics - A review. Lait. 1995;75(6):447–452.

202. Zarate G. Dairy Propionibacteria: Less Conventional Probiotics to Improve the Human and Animal Health. Probiotic Anim. 2012.

203. Trottmann F, Franke J, Richter I, Ishida K, Cyrulies M, Dahse HM, et al. Cyclopropanol Warhead in Malleicyprol Confers Virulence of Human‐ and Animal‐Pathogenic Burkholderia Species. Angew Chem Int Ed. 2019;58(40):14129–33.

204. Sadiq MA, Hassan L, Aziz SA, Zakaria Z, Musa HI, Amin MM, et al. Phylogenetic Diversity of Burkholderia pseudomallei isolated from veterinary cases and the environments in Peninsular Malaysia. Vet Anim Sci. 2018;6:21–8.

205. Fonseca Júnior AA, Pinto CA, Alencar CAdS, Bueno BL, dos Reis JKP, de Carvalho Filho MB. Validation of three qPCR for the detection of Burkholderia mallei in equine tissue samples. Arch Microbiol. 2021;203(7):3965–71.

206. Wooten RM, Jelesijevic T, Zimmerman SM, Harvey SB, Mead DG, Shaffer TL, et al. Use of the Common Marmoset to Study Burkholderia mallei Infection. PLoS ONE. 2015;10(4).

207. Dance DAB. Ecology of Burkholderia pseudomallei and the interactions between environmental Burkholderia spp. and human–animal hosts. Acta Trop. 2000;74(2-3):159–68.

208. Daniel SL, Moradi L, Paiste H, Wood KD, Assimos DG, Holmes RP, et al. Forty Years of Oxalobacter formigenes, a Gutsy Oxalate-Degrading Specialist. Appl Environ Microbiol. 2021;87(18).

209. Stewart CS, Duncan SH, Cave DR. Oxalobacter formigenes and its role in oxalate metabolism in the human gut. FEMS Microbiol Lett. 2004;230(1):1–7.

210. Weese JS, Weese HE, Rousseau J. Identification of Oxalobacter formigenes in the faeces of healthy cats. Lett Appl Microbiol. 2009;49(6):800–2.

211. Miller AW, Dale C, Dearing MD, Langille MGI. The Induction of Oxalate Metabolism In Vivo Is More Effective with Functional Microbial Communities than with Functional Microbial Species. mSystems. 2017;2(5).

212. Sellera FP, Fernandes MR, Fuga B, Fontana H, Vásquez-Ponce F, Goldberg DW, et al. Phylogeographical Landscape of Citrobacter portucalensis Carrying Clinically Relevant Resistomes. Microbiol Spectr. 2022;10(2).

213. Ribeiro TG, Gonçalves BR, da Silva MS, Novais Â, Machado E, Carriço JA, et al. Citrobacter portucalensis sp. nov., isolated from an aquatic sample. Int J Syst Evol Microbiol. 2017;67(9):3513–7.

214. Diaz OL, Buendia A, Sánchez J, Villalobos G, Rojas-Serrania N, Cervantes JAO, et al. Identification of enterobacteriaceae causing septicemia in the axolotl Ambystoma mexicanum. Antonie van Leeuwenhoek. 2025;118(3).

215. Thomas SG, Abajorga M, Glover MA, Wengert PC, Parthasarathy A, Savka MA, et al. Aeromonas hydrophila RIT668 and Citrobacter portucalensis RIT669—Potential Zoonotic Pathogens Isolated from Spotted Turtles. Microorganisms. 2020;8(11).

216. Santagati M, Campanile F, Stefani S. Genomic Diversification of Enterococci in Hosts: The Role of the Mobilome. Front Microbiol. 2012;3.

217. Byappanahalli MN, Nevers MB, Korajkic A, Staley ZR, Harwood VJ. Enterococci in the Environment. Microbiol Mol Biol Rev. 2012;76(4):685–706.

218. Murray BE. The Life and Times of the Enterococcus. Clin Microbiol Rev. 1990;3(1):46–65.

219. Romalde JL, Magariños B, Núñez S, Barja JL, Toranzo AE. Host Range Susceptibility of Enterococcus sp. Strains Isolated from Diseased Turbot: Possible Routes of Infection. Appl Environ Microbiol. 1996;62(2):607–611.

220. Gülhan T, Boynukara B, Durmuş A, Kiziroğlu İ, Sancak YC. Enteric bacteria and some pathogenic properties of Enterococcus faecalis, Enterococcus faecium and Escherichia coli strains isolated from wild ducks and gulls. Fresenius Environ Bull. 2012;21(7a):1961–1966.

221. Singh, Vikash. Salmonella Serovars and Their Host Specificity. J Vet Sci Anim Husb. 2013;1(3).

222. Kaiser P, Rothwell L, Galyov EE, Barrow PA, Burnside J, Wigley P. Differential cytokine expression in avian cells in response to invasion by Salmonella typhimurium, Salmonella enteritidis and Salmonella gallinarum. Microbiology. 2000;146:3217–3226.

223. Putturu R, Eevuri T, Ch B, Nelapati K. Salmonella enteritidis – Food Borne Pathogen – A Review. Int J Pharm Bio Sci. 2015;5(1):86–95.

224. Langridge GC, Fookes M, Connor TR, Feltwell T, Feasey N, Parsons BN, et al. Patterns of genome evolution that have accompanied host adaptation in Salmonella. Proc Natl Acad Sci USA. 2014;112(3):863–8.

225. Novoslavskij A, Terentjeva M, Eizenberga I, Valciņa O, Bartkevičs V, Bērziņš A. Major foodborne pathogens in fish and fish products: a review. Ann Microbiol. 2015;66(1):1–15.

226. Mohan K, Muvavarirwa P, Pawandiwa A. Strains of Actinobacillus spp. from diseases of animals and ostriches in Zimbabwe. Onderstepoort J Vet Res. 1997;64:195–199.

227. Dawson JD, Cockel R. Oesophageal perforation at fibreoptic gastroscopy. Br Med J. 1981;283:583.

228. Sassu EL, Bossé JT, Tobias TJ, Gottschalk M, Langford PR, Hennig-Pauka I. Update on Actinobacillus pleuropneumoniae-knowledge, gaps and challenges. Transbound Emerg Dis. 2018;65:72–90.

229. Taichman NS, Simpson DL, Sakurada S, Cranfield M, DiRienzo J. Comparative studies on the biology of Actinobacillus actinomycetemcomitans leukotoxin in primates. Oral Microbiol Immunol. 1987;2(3):97-104.

230. De Koster S, Rodriguez Ruiz JP, Rajakani SG, Lammens C, Glupczynski Y, Goossens H, et al. Diversity in the Characteristics of Klebsiella pneumoniae ST101 of Human, Environmental, and Animal Origin. Front Microbiol. 2022;13.

231. Wareth G, Neubauer H. The Animal-foods-environment interface of Klebsiella pneumoniae in Germany: an observational study on pathogenicity, resistance development and the current situation. Vet Res. 2021;52(1).

232. Harada K, Shimizu T, Mukai Y, Kuwajima K, Sato T, Usui M, et al. Phenotypic and Molecular Characterization of Antimicrobial Resistance in Klebsiella spp. Isolates from Companion Animals in Japan: Clonal Dissemination of Multidrug-Resistant Extended-Spectrum β-Lactamase-Producing Klebsiella pneumoniae. Front Microbiol. 2016;7:1021.

233. Proietti PC, Passamonti F, Franciosini MP, Asdrubali G. Hafnia alvei infection in pullets in Italy. Avian Pathol. 2010;33(2):200–4.

234. Valiev A, Ermakov V, Titov N, Ziganshin B, Nezhmetdinova F, Taylan A, et al. Persistence factors and antibiotic susceptibility of enterobacteria isolated from various animal species. BIO Web Conf. 2022;52.

235. Petchell WHR, Noble P-JM, Burrow R, Humphreys WJE, Díaz-Delgado OB. Hafnia alvei: The unreported pathogen responsible for a sub-capsular renal abscess in a 1-year-old, presumed immunocompetent crossbreed dog with no co-morbidities. Vet Rec Case Rep. 2021;9:e55.

236. Zhang Q, Han S, Liu K, Luo J, Lu J, He H. Occurrence of Selected Zoonotic Fecal Pathogens and First Molecular Identification of Hafnia paralvei in Wild Taihangshan Macaques (Macaca mulatta tcheliensis) in China. BioMed Res Int. 2019;2019:1–7.

237. Pinnell LJ, Reyes AA, Wolfe CA, Weinroth MD, Metcalf JL, Delmore RJ, et al. Bacteroidetes and Firmicutes Drive Differing Microbial Diversity and Community Composition Among Micro-Environments in the Bovine Rumen. Front Vet Sci. 2022;9.

238. Turner PV. The role of the gut microbiota on animal model reproducibility. Anim Models Exp Med. 2018;1(2):109–15.

239. O’ Donnell MM, Harris HMB, Ross RP, O'Toole PW. Core fecal microbiota of domesticated herbivorous ruminant, hindgut fermenters, and monogastric animals. MicrobiologyOpen. 2017;6(5).

240. Suchodolski JS. COMPANION ANIMALS SYMPOSIUM: Microbes and gastrointestinal health of dogs and cats1. J Anim Sci. 2011;89(5):1520–30.

241. Eeckhaut V, Van Immerseel F, Croubels S, De Baere S, Haesebrouck F, Ducatelle R, et al. Butyrate production in phylogenetically diverse Firmicutes isolated from the chicken caecum. Microbial Biotechnology. 2011;4(4):503–12.

242. Fernández Lanza V, Tedim AP, Martínez JL, Baquero F, Coque TM. The Plasmidome of Firmicutes: Impact on the Emergence and the Spread of Resistance to Antimicrobials. Microbiol Spectrum. 2015;3(2):PLAS-0039-2014.

243. Degregori S, Johnson GC, Barber PH, Blumstein DT, Flaherty E. Firmicutes and Bacteroidetes contribute to mass gain variation in female obligate hibernators. J Mammal. 2024;105(1):2–12.

244. Hanning I, Diaz-Sanchez S. The functionality of the gastrointestinal microbiome in non-human animals. Microbiome. 2015;3(1).

245. Bensch HM, Tolf C, Waldenström J, Lundin D, Zöttl M. Bacteroidetes to Firmicutes: captivity changes the gut microbiota composition and diversity in a social subterranean rodent. Anim Microbiome. 2023;5(1).

246. Vancanneyt M, Segers P, Hauben L, Hommez J, Devriese LA, Hoste B, et al. Flavobacterium meningosepticum, a Pathogen in Birds. J Clin Microbiol. 1994;32(10):2398–2403.

247. Verma DK, Rathore G. New host record of five Flavobacterium species associated with tropical fresh water farmed fishes from North India. Braz J Microbiol. 2015;46(4):969–76.

248. Welker TL, Shoemaker CA, Arias CR, Klesius PH. Transmission and detection of Flavobacterium columnare in channel catfish Ictalurus punctatus. Dis Aquat Org. 2005;63(2-3):129-138.

249. LaFrentz BR, García JC, Waldbieser GC, Evenhuis JP, Loch TP, Liles MR, et al. Identification of Four Distinct Phylogenetic Groups in Flavobacterium columnare With Fish Host Associations. Front Microbiol. 2018;9.

250. Haenni M, Hocquet D, Ponsin C, Cholley P, Guyeux C, Madec J-Y, et al. Population structure and antimicrobial susceptibility of Pseudomonas aeruginosa from animal infections in France. BMC Vet Res. 2015;11(1).

251. Mushin R, Ziv G. An epidemiological study of Pseudomonas aeruginosa in cattle and other animals by pyocine typing. J Hyg. 2009;71(1):113–22.

252. Moura-Alves P, Puyskens A, Stinn A, Klemm M, Guhlich-Bornhof U, Dorhoi A, et al. Host monitoring of quorum sensing during Pseudomonas aeruginosa infection. Science. 2019;366(6472).

253. Duan K, Dammel C, Stein J, Rabin H, Surette MG. Modulation of Pseudomonas aeruginosa gene expression by host microflora through interspecies communication. Mol Microbiol. 2003;50(5):1477–91.

254. Kim M, Lee J-H, Kim E, Choi H, Kim Y, Lee J. Isolation of Indole Utilizing Bacteria Arthrobacter sp. and Alcaligenes sp. From Livestock Waste. Indian J Microbiol. 2016;56(2):158–66.

255. Fitriyanto NA, Natalia D, Prasetyo RA, Erwanto Y, Panjono, Ngadiono N. Properties of rabbit feces composting using indigenous Alcaligenes sp. LS2T and Arthrobacter sp. LM1KK. IOP Conf Ser Earth Environ Sci. 2021;662(1):012014.

256. El-Jakee J, Nagwa AS, Bakry M, Zouelfakar SA, Elgabry E, Gad El-Said WA. Characteristics of Staphylococcus aureus Strains Isolated from Human and Animal Sources. Am-Euras J Agric Environ Sci. 2008;4(2):221–229.

257. Reizner W, Hunter JG, O’Malley NT, Southgate RD, Schwarz EM, Kates SL. A systematic review of animal models for Staphylococcus aureus osteomyelitis. Eur Cell Mater. 2014;27:196–212.

258. Leonard FC, Markey BK. Meticillin-resistant Staphylococcus aureus in animals: A review. Vet J. 2008;175(1):27–36.

259. de Lencastre H, Monecke S, Gavier-Widén D, Hotzel H, Peters M, Guenther S, et al. Diversity of Staphylococcus aureus Isolates in European Wildlife. PLoS ONE. 2016;11(12).

260. Porrero MC, Mentaberre G, Sánchez S, Fernández-Llario P, Casas-Díaz E, Mateos A, et al. Carriage of Staphylococcus aureus by Free-Living Wild Animals in Spain. Appl Environ Microbiol. 2014;80(16):4865–70.

261. Colles FM, Jones K, Harding RM, Maiden MCJ. Genetic Diversity of Campylobacter jejuni Isolates from Farm Animals and the Farm Environment. Appl Environ Microbiol. 2003;69(12):7409–13.

262. Manser PA, Dalziel RW. A survey of campylobacter in animals. J Hyg. 2009;95(1):15–21.

263. Sangioni LA, Horta MC, Vianna MCB, Gennari SM, Soares RM, Galvão MAM, et al. Food and animal sources of human Campylobacter jejuni infection. Emerg Infect Dis. 2005;11(2):265–70.

264. Nomoto R, Hong Thuy Tien L, Sekizaki T, Osawa R. Antimicrobial Susceptibility of Streptococcus gallolyticus Isolated from Humans and Animals. Jpn J Infect Dis. 2013;66(4):334–336.

265. Vela AI, Villalón P, Sáez-Nieto JA, Chacón G, Domínguez L, Fernández-Garayzábal JF. Characterization of Streptococcus pyogenes from Animal Clinical Specimens, Spain. Emerg Infect Dis. 2017;23(12):2013–6.

266. Timoney JF. Streptococcus. Pathogenesis of Bacterial Infections in Animals 2022. p. 565–87.

267. Goh SH, Driedger D, Gillett S, Low DE, Hemmingsen SM, Amos M, et al. Streptococcus iniae, a human and animal pathogen: specific identification by the chaperonin 60 gene identification method. J Clin Microbiol. 1998;36(7):2164–2166.

268. Cortez Nunes F, Letra Mateus T, Taillieu E, Teixeira S, Carolino N, Rema A, et al. Molecular detection of Helicobacter spp. and Fusobacterium gastrosuis in pigs and wild boars and its association with gastric histopathological alterations. Vet Res. 2022;53(1).

269. Morales B AA, García G F, Bermúdez G VM. El Género Helicobacter en los animales domésticos: Una Revisión. Revista del Instituto Nacional de Higiene Rafael Rangel. 2010;41(2):1–8.

270. Taillieu E, Chiers K, Amorim I, Gärtner F, Maes D, Van Steenkiste C, et al. Gastric Helicobacter species associated with dogs, cats and pigs: significance for public and animal health. Vet Res. 2022;53(1).

271. Uddin W, Khan G, Narayan S, Sharma N, Holeyachi BS, Hakeem MA, et al. Prevalence and diversity of Helicobacter species in captive wild carnivores, and their implications for conservation management of endangered species. BMC Vet Res. 2025;21(1):498.

272. Oxley APA, McKay DB. Comparison of Helicobacter spp. genetic sequences in wild and captive seals, and gulls. Dis Aquat Org. 2005;65:99–105.

273. De Witte C, Lemmens C, Flahou B, De Laender P, Bouts T, Vercammen F, et al. Presence of Helicobacter and Campylobacter species in faecal samples from zoo mammals. Vet Microbiol. 2018;219:49–52.

274. C NF. Presence of Helicobacter Spp. And Fusobacterium Gastrosuis in the Stomach of Domestic and Wild Animals An Epidemiological and Molecular Study.2023.

275. Angelos JA, Clothier KA, Agulto RL, Mandzyuk B, Tryland M. Relatedness of type IV pilin PilA amongst geographically diverse Moraxella bovoculi isolated from cattle with infectious bovine keratoconjunctivitis. J Med Microbiol. 2021;70(2).

276. Embers ME, Doyle LA, Whitehouse CA, Selby EB, Chappell M, Philipp MT. Characterization of a Moraxella species that causes epistaxis in macaques. Vet Microbiol. 2011;147(3-4):367–75.

277. Kodjo A, Moussa A, Borges E, Richard Y. Identification of Moraxella‐like Bacteria Isolated from Caprine and Ovine Nasal Flora. J Vet Med B. 2010;40(1-10):97–104.

278. Kim K-T, Lee S-H, Kwak D. Identification of Moraxella lacunata from pulmonary abscesses in three zoo herbivores. J Vet Med Sci. 2018;80(12):1914–7.

279. Pugh GW Jr, Hughes DE, Schulz VD. The Pathophysiological Effects of Moraxella bovis Toxins on Cattle, Mice and Guinea Pigs. Can J Comp Med. 1973;37(1):70–78.

280. Patrick S. A tale of two habitats: Bacteroides fragilis, a lethal pathogen and resident in the human gastrointestinal microbiome. Microbiology. 2022;168(4).

281. Gómez‐Doñate M, Payán A, Cortés I, Blanch AR, Lucena F, Jofre J, et al. Isolation of bacteriophage host strains of Bacteroides species suitable for tracking sources of animal faecal pollution in water. Environ Microbiol. 2011;13(6):1622–31.

282. Leser TD, Mølbak L. Better living through microbial action: the benefits of the mammalian gastrointestinal microbiota on the host. Environ Microbiol. 2009;11(9):2194–206.

283. Kollarcikova M, Faldynova M, Matiasovicova J, Jahodarova E, Kubasova T, Seidlerova Z, et al. Different Bacteroides Species Colonise Human and Chicken Intestinal Tract. Microorganisms. 2020;8(10).

284. Chi X, Gao H, Wu G, Qin W, Song P, Wang L, et al. Comparison of gut microbiota diversity between wild and captive bharals (Pseudois nayaur). BMC Vet Res. 2019;15(1).

285. Aworh MK, Chalmers G, Anderson REV, Murray R, Topp E, Boerlin P. Characterization of Proteus mirabilis and associated plasmids isolated from anaerobic dairy cattle manure digesters. PLoS ONE. 2023;18(8).

286. Marques C, Belas A, Menezes J, Moreira da Silva J, Cavaco-Silva P, Trigueiro G, et al. Human and Companion Animal Proteus mirabilis Sharing. Microbiol Res. 2021;13(1):38–48.

287. Kusumoto M, Kanao Y, Narita H, Jitsuiki M, Iyori K, Tsunoi M, et al. In vitro efficacy of cephamycins against multiple extended-spectrum β-lactamase producing Klebsiella pneumoniae, Proteus mirabilis, and Enterobacter cloacae isolates from dogs and cats. J Vet Med Sci. 2023;85(6):653–6.

288. Sanches MS, Baptista AAS, de Souza M, Menck-Costa MF, Justino L, Nishio EK, et al. Proteus mirabilis causing cellulitis in broiler chickens. Braz J Microbiol. 2020;51(3):1353–62.

289. Bautista E, Martino P, Manacorda A, Cossu ME, Stanchi N. Spontaneous Proteus mirabilis and Enterobacter aerogenes infection in chinchilla (Chinchilla lanigera). Scientifur. 2007;31(1):27-29.

290. Lv P, Hao G, Cao Y, Cui L, Wang G, Sun S. Detection of Carbapenem Resistance of Proteus mirabilis Strains Isolated from Foxes, Raccoons and Minks in China. Biology. 2022;11(2).

291. Tsai MA, Wang PC, Liaw LL, Yoshida T, Chen SC. Comparison of genetic characteristics and pathogenicity of Lactococcus garvieae isolated from aquatic animals in Taiwan. Dis Aquat Org. 2012;102(1):43–51.

292. Kawanishi M, Yoshida T, Yagashiro S, Kijima M, Yagyu K, Nakai T, et al. Differences between Lactococcus garvieae isolated from the genus Seriola in Japan and those isolated from other animals (trout, terrestrial animals from Europe) with regard to pathogenicity, phage susceptibility and genetic characterization. J Appl Microbiol. 2006;101(2):496–504.

293. Thiry D, Billen F, Boyen F, Duprez J-N, Quenault H, Touzain F, et al. Genomic relatedness of a canine Lactococcus garvieae to human, animal and environmental isolates. Res Vet Sci. 2021;137:170–3.

294. Brzóska F, Śliwiński B, Stecka K. Effect of Lactococcus lactis vs. Lactobacillus spp. bacteria on chicken body weight, mortality, feed conversion and carcass quality. Ann Anim Sci. 2012;12(4):549–59.

295. Pot B, Devriese LA, Ursi D, Vandamme P, Haesebrouck F, Kersters K. Phenotypic identification and differentiation of Lactococcus strains isolated from animals. Syst Appl Microbiol. 1996;19(2):213–22.

296. Gurien LA, Stallings-Archer K, Smith SD. Probiotic Lactococcus lactis decreases incidence and severity of necrotizing enterocolitis in a preterm animal model. J Neonatal-Perinat Med. 2018;11(1):65–9.

297. Uzal FA, McClane BA, Cheung JK, Theoret J, Garcia JP, Moore RJ, et al. Animal models to study the pathogenesis of human and animal Clostridium perfringens infections. Vet Microbiol. 2015;179(1-2):23–33.

298. Janezic S, Zidaric V, Pardon B, Indra A, Kokotovic B, Blanco JL, et al. International Clostridium difficile animal strain collection and large diversity of animal associated strains. BMC Microbiol. 2014;14:173.

299. Arroyo LG, Kruth SA, Willey BM, Staempfli HR, Low DE, Weese JS. PCR ribotyping of Clostridium difficile isolates originating from human and animal sources. J Med Microbiol. 2005;54(2):163–6.

300. Immerseel FV, Buck JD, Pasmans F, Huyghebaert G, Haesebrouck F, Ducatelle R. Clostridium perfringens in poultry: an emerging threat for animal and public health. Avian Pathol. 2004;33(6):537–49.

301. Shayegani M, DeForge I, McGlynn DM, Root T. Characteristics of Yersinia enterocolitica and related species isolated from human, animal, and environmental sources. J Clin Microbiol. 1981;14(3):304–312.

302. Bari ML, Hossain MA, Isshiki K, Ukuku D. Behavior of Yersinia enterocolitica in Foods. J Pathog. 2011;2011:1–13.

303. Lin B, Syczyło K, Platt-Samoraj A, Bancerz-Kisiel A, Szczerba-Turek A, Pajdak-Czaus J, et al. The prevalence of Yersinia enterocolitica in game animals in Poland. PLoS ONE. 2018;13(3).

304. Liang J, Zhu Z, Lan R, Meng J, Vrancken B, Lu S, et al. Evolutionary and genomic insights into the long-term colonization of Shigella flexneri in animals. Emerg Microbes Infect. 2022;11(1):2069–79.

305. Morse EV, Duncan MA. Canine Salmonellosis: Prevalence, Epizootiology, Signs, and Public Health Significance. J Am Vet Med Assoc. 1975;167(9):817–820.

306. Ojcius DM, Shi R, Yang X, Chen L, Chang H-t, Liu H-y, et al. Pathogenicity of Shigella in Chickens. PLoS ONE. 2014;9(6).

307. Kim YJ, Yeo SG, Park JH, Ko HJ. Shigella Vaccine Development: Prospective Animal Models and Current Status. Curr Pharm Biotechnol. 2013;14(10):903–912.

308. Lampel KA, Formal† SB, Maurelli AT, Kaper J. A Brief History of Shigella. EcoSal Plus. 2018;8(1).

309. O'Donnell K, Sutton DA, Rinaldi MG, Sarver BAJ, Balajee SA, Schroers H-J, et al. Internet-Accessible DNA Sequence Database for Identifying Fusaria from Human and Animal Infections. J Clin Microbiol. 2010;48(10):3708–18.

310. Thrane U. Fusarium. Encyclopedia of Food Microbiology.2014. p. 76–81.

311. Antonissen G, Martel A, Pasmans F, Ducatelle R, Verbrugghe E, Vandenbroucke V, et al. The Impact of Fusarium Mycotoxins on Human and Animal Host Susceptibility to Infectious Diseases. Toxins. 2014;6(2):430–52.

312. Forster RK, Rebell G. Animal model of Fusarium solani keratitis. Am J Ophthalmol. 1975;79(3):510–515.

313. Yu J, Cleveland TE, Nierman WC, Bennett JW. Aspergillus flavus genomics: gateway to human and animal health, food safety, and crop resistance to diseases. Rev Iberoam Micol. 2005;22:194–202.

314. Pena GA, Pereyra CM, Armando MR, Chiacchiera SM, Magnoli CE, Orlando JL, et al. Aspergillus fumigatus toxicity and gliotoxin levels in feedstuff for domestic animals and pets in Argentina. Lett Appl Microbiol. 2010;50(1):77–81.

315. de Jong JE, Heuvelink AE, Dieste Pérez L, Holstege MMC. Aspergillus spp., aspergillosis and azole usage in animal species in Europe: Results from a multisectoral survey and review of recent literature. Med Mycol. 2025;63(2).

316. Lee S-K, Park S-Y, Kang H-Y, Han S-J, Nam H-Y, Choi C-Y, et al. Prevalence of the Cladosporium cladosporioides Species Complex in the Mycelia-Like Skin Crusts of Migratory Yellow-Throated Buntings (Emberiza elegans) in Korea. Mycopathologia. 2025;190(2).

317. Sandoval-Denis M, Sutton DA, Martin-Vicente A, Cano-Lira JF, Wiederhold N, Guarro J, et al. Cladosporium Species Recovered from Clinical Samples in the United States. J Clin Microbiol. 2015;53(9):2990–3000.

318. Spano M, Zuliani D, Peano A, Bertazzolo W. Cladosporium cladosporioides‐complex infection in a mixed‐breed dog. Vet Clin Pathol. 2018;47(1):150–3.
